# Identification of Four Novel Prognostic Biomarkers and Construction of Two Nomograms in Adrenocortical Carcinoma: A Multi-Omics Data Study *via* Bioinformatics and Machine Learning Methods

**DOI:** 10.3389/fmolb.2022.878073

**Published:** 2022-05-25

**Authors:** Xiaochun Yi, Yueming Wan, Weiwei Cao, Keliang Peng, Xin Li, Wangchun Liao

**Affiliations:** Department of Urology, Yueyang People’s Hospital, Hunan Normal University, Yueyang, China

**Keywords:** adrenocortical carcinoma, WGCNA, hub genes, nomogram, prognosis, immune microenvironment, copy number variations

## Abstract

**Background:** Adrenocortical carcinoma (ACC) is an orphan tumor which has poor prognoses. Therefore, it is of urgent need for us to find candidate prognostic biomarkers and provide clinicians with an accurate method for survival prediction of ACC via bioinformatics and machine learning methods.

**Methods:** Eight different methods including differentially expressed gene (DEG) analysis, weighted correlation network analysis (WGCNA), protein-protein interaction (PPI) network construction, survival analysis, expression level comparison, receiver operating characteristic (ROC) analysis, and decision curve analysis (DCA) were used to identify potential prognostic biomarkers for ACC via seven independent datasets. Linear discriminant analysis (LDA), K-nearest neighbor (KNN), support vector machine (SVM), and time-dependent ROC were performed to further identify meaningful prognostic biomarkers (MPBs). Cox regression analyses were performed to screen factors for nomogram construction.

**Results:** We identified nine hub genes correlated to prognosis of patients with ACC. Furthermore, four MPBs (ASPM, BIRC5, CCNB2, and CDK1) with high accuracy of survival prediction were screened out, which were enriched in the cell cycle. We also found that mutations and copy number variants of these MPBs were associated with overall survival (OS) of ACC patients. Moreover, MPB expressions were associated with immune infiltration level. Two nomograms [OS-nomogram and disease-free survival (DFS)-nomogram] were established, which could provide clinicians with an accurate, quick, and visualized method for survival prediction.

**Conclusion:** Four novel MPBs were identified and two nomograms were constructed, which might constitute a breakthrough in treatment and prognosis prediction of patients with ACC.

## Introduction

Though adrenocortical carcinoma (ACC) is an uncommon malignancy, the prognosis of patients with this malignancy is poor (Jasim and Habra, 2019). The disease tends to occur in 3 to 4-year-old children and 40 to 50-year-old adults (Libé, 2018). The incidence of ACCs in children is reported to be as low as 0.2% of pediatric cancers (Libé, 2018). As a recent study reported, there were 15,800 new cases of ACC worldwide in 2018 (Bray et al., 2018). However, the incidence of ACCs varies from place to place around the world (Bray et al., 2018). In some countries, such as southern Brazil, the incidence is 10–15 times what it is in America (Bray et al., 2018). But the consensus is that this malignancy heavily endangers health and is very difficult to cure (Lo et al., 2019). According to the previous studies, some researchers tried to diagnose ACC earlier to grasp the optimal treatment opportunity (Lo et al., 2019). What is worse, about 40 percent of ACCs had distant metastasis when they were diagnosed (Guillaume et al., 2014). Nowadays, the discoveries of new small biomarkers greatly aid diagnosis of malignant tumors by using the methods of molecular biology and bioinformatics (He et al., 2017). In order to better diagnosis ACCs and improve the prognosis of patients, the objective of the research is to screen several effective prognostic biomarkers of ACC. Also, we attempted to provide clinicians with several choices for ACC therapy. The CMap analysis demonstrated that five small molecule drugs including chlorpromazine, trifluoperazine, alpha-estradiol, 15-delta prostaglandin J2, and vorinostat might be novel drugs for ACC treatment. These MPBs were also significantly enriched in the cell cycle. As for the enriched drugs, ASPM was significantly enriched in 6 drugs, BIRC5 was associated with 6 drugs, CCNB2 was related to 11 drugs, and CDK1 was enriched in 6 drugs. Moreover, we devoted ourselves to provide clinicians with an accurate, individual, and visualized method to predict overall survival (OS) or disease-free survival (DFS) of patients with ACC. To do this, we thought it could help clinicians to understand and master the illness and better formulate the treatment scheme.

## Methods and Materials

### ACC Microarray Studies Identification

All the GEO datasets were downloaded from the GEO database (http://www.ncbi.nlm.nih.gov/geo/). For differentially expressed gene (DEG) screening, datasets with related control tissues were collected and used. Then two datasets including GSE75415 (West et al., 2007) and GSE12368 (Soon et al., 2009) were included. Then four datasets including GSE76021 (Pinto et al., 2016), GSE19750 (Demeure et al., 2013), GSE10927 (Giordano et al., 2009), and GSE76019 (Surakhy et al., 2020) from this database were collected and used in the present study, because of the complete clinical and survival information they contained. Moreover, we retrieved microarray data of ACC (TCGA-ACC data) and the related clinical information via The Cancer Genome Atlas (TCGA) database (https://genome-cancer. ucsc.edu/). All in all, GSE12368 and GSE75415 were included for DEG identification because they included normal tissues. GSE76021, GSE19750, GSE10927, GSE76019, and TCGA-ACC data were included because they had related clinical information (stage, grade, etc.) and survival information. The details of all the datasets are shown in Supplementary Table S1.

### Data Preprocessing and DEG Identification

For the TCGA data, we firstly downloaded RNA sequencing data (FPKM value) of gene expressions from the TCGA database using R package “TCGAbiolinks” (Colaprico et al., 2016). In order to compare and validate the results with GEO datasets, these data were further transformed into a transcripts per kilobase million (TPM) profile. For the datasets from the GEO database, the robust multichip average algorithm (Irizarry et al., 2003) was used because the data were displayed as RAW series. Moreover, log2 transformation and normalization were conducted based on R package “affy” (Gautier et al., 2004).

How we validated the results among this study is shown in Supplementary Figure S1. A total of 29 ACCs included in GSE76021 were used for WGCNA. We sorted genes according to their variance across all samples, all genes were selected for WGCNA. Moreover, differentially expressed genes (DEGs) between ACCs and normal tissues were filtered out by the criterion (*p* value < 0.05, |log2 fold change (FC) | ≥ 1.5) via R package “limma” (Ritchie et al., 2015) for further study. Then DEGs overlapped between GSE75415 and GSE12368 were screened for subsequent analysis.

### Co-Expression Network Construction

Before conducting WGCNA, the expression matrix of the transcript level was checked via two approaches (goodSamplesGenes and sample network methods) in R package “WGCNA” (Zhang and Horvath, 2005). Only samples of Z.Ku ≥ -2.5 were included for co-expression network construction. By means of the scale free topology criterion, β (soft threshold power beta) was chosen. We subsequently transformed adjacency into TOM. Then based on the TOM, genes were classified into modules via the branch cutting approach. Some important parameters set in the present study were shown as below: minClusterSize = 30, deepSplit = 2. In addition, by selecting a cut line reckoned dissimilarity of module eigengenes (MEs), modules showing high correlation with each other were merged.

### Survival-Associated Module Identification

After determining modules composed of genes, two methods were applied on screening hub modules which were relevant to survival status (the aimed clinical trait). The correlation between module eigengenes and traits were quantized. Next, through evaluating gene significance (GS), the relationship between genes and traits was measured. In addition, the average GS of all the genes in a module was further worked out, which represented the module significance (MS). After finishing the above analyses, we identified the most related module as the key module.

### Connectivity Map Analysis

As a convenient webtool, researchers can quickly locate molecule drugs which have potential against related diseases through CMap (https://portals.broadinstitute.org/cmap/) (Lamb et al., 2006). Therefore, CMap analysis was conducted via the screened DEGs, in order to explore potential drugs showing a strong relationship with ACC. Drugs meeting the requirement [number of instances (n) > 10, *p* value < 0.05] were considered significant. Furthermore, drugs with |mean| ≥ 0.40 were further screened out, which might be useful choices for treating ACC.

### Candidate Hub Gene Construction

After choosing the key module, genes of |cor.geneModuleMembership| >0.8 and |cor.geneTraitSignificance| >0.2 were regarded as hub genes in WGCNA. Then we constructed a protein-protein interaction (PPI) network of these genes via the Search Tool for the Retrieval of Interacting Genes (STRING) (Szklarczyk et al., 2015). The following parameters were important and listed: network scoring: degree cutoff = 2; cluster finding: node score cutoff = 0.2, k-core = 2, and max. depth = 100. A vehicle named network analyzer in Cytoscape (Shannon et al., 2003) was used for the gene degree of connectivity calculation. In this research, we regarded a gene as a hub gene in the PPI network when its degree ≥4. We also constructed a PPI network for DEGs to screen hub genes in DEGs by using the same standard. Finally, genes overlapping between hub genes in WGCNA and hub genes in DEGs were considered as candidate hub genes, which were included for further analysis. Gene ontology (GO) (Ashburner et al., 2000) enrichment analysis and Kyoto Encyclopedia of Genes and Genomes (KEGG) (Kanehisa and Goto, 2000) pathway analysis were conducted via R package “clusterProfiler” (Yu et al., 2012) for functional annotation of candidate hub genes. We selected *p* < 0.05 as the standards to define significant BPs and KEGG pathway terms.

### Hub Gene Identification

Hub genes related to survival and prognosis of ACC patients were screened through performing survival analysis among candidate hub genes based on R package “survival” (Therneau, 2015) for datasets with complete survival information (GSE19750, GSE76019, GSE76021, and TCGA-ACC data). For TCGA-ACC data, 79 samples with complete overall survival (OS) information were included for OS analysis, meanwhile 54 samples with complete disease-free survival (DFS) information were included for DFS analysis. According to the candidate hub gene expression levels, we split samples into two groups (high expression group and low expression group) in all the datasets (the median expression of each candidate hub gene in each dataset was set as the grouping standard). Genes of *p* < 0.05 in all survival analyses were considered as hub genes.

### Hub Gene Validation

Based on datasets with complete stage information (GSE10927, GSE19750, GSE75415, GSE76019, GSE76021 and TCGA-ACC data), we plotted tumor stage (I, II, III and IV) boxplots using the “ggstatsplot” (Patil, 2018) R package. Moreover, tumor grade boxplots were also plotted based on GSE10927 (low grade and high grade) and GSE19750 (grade 1, grade 2, grade 3, and grade 4). A one-way analysis of variance (ANOVA) test was conducted to evaluate the results when samples were divided into more than two groups. We used unpaired *t* test to measure the statistical significance when samples were divided into two groups. Moreover, the difference of hub gene expression values in ACCs, ACAs, and normal adrenal samples were measured using GSE10927, GSE12368, GSE19750, GSE75415, and TCGA-BLCA data.

### Receiver Operating Characteristic Analysis and Decision Curve Analysis

Through R package “plotROC” (Sachs, 2017), ROC curve analysis was performed. In GSE10927, GSE12368, GSE19750, and GSE75415, the AUC was calculated to differentiate ACC samples and normal tissues. In GSE10927, GSE19750, GSE75415, GSE76019, GSE76021, and TCGA-ACC data, we worked out the AUC to distinguish localized ACC and advanced ACC. In this study, we regarded ACC of stages I or II as localized ACC and ACC of stages III or IV as advanced ACC. In both GSE10927 and GSE19750, we worked out the AUC to distinguish ACC of low grade (grades 1 or 2) and ACC of high grade (grades 3 or 4). Moreover, we distinguished ACA and ACC in GSE10927, GSE12368, and GSE75415. In this study, we thought genes could distinguish ACC samples from normal tissues (localized ACC from advanced ACC or low grade ACC from high grade ACC) well when the AUC was more than 0.70. Furthermore, DCA (Vickers and Elkin, 2006) was performed for verifying the hub genes’ diagnostic potential by using GSE76021.

### Linear Discriminant Analysis, K-Nearest Neighbor, and Support Vector Machine to Screen Genes With High Accuracy of Predicting OS Among Hub Genes

To validate hub genes’ prognostic potential, genes were taken as variables, relative mRNA expression values of which were taken as variable values. LDA, KNN, and SVM analyses were immediately conducted. LDA was conducted via R package “MASS” (Venables and Ripley, 2002). The cross validation approach was used to pick out the best K parameter via R package “caret” (Kuhn, 2015). Based on the best K parameter, R packages “class” (Venables and Ripley, 2002) and “kknn” were used for the KNN method. In addition, we performed four types of SVM methods via R package “e1071”. They were linear-SVM, polynomial-SVM, radial basis function (RBF) SVM, and sigmoid-kernel SVM, separately. The SVM factors setting was based on “kernlab” in R software. TCGA-ACC data were included in this part. We regarded a gene as a meaningful prognostic biomarker (MPB) with the average accuracy of classification in three analyses ≥0.80.

### Time-Dependent ROC Analysis for MPBs

To verify the potential of the prognosis prediction of MPBs, based on TCGA-ACC data, time-independent (1-, 3-, 5-years) receiver operating characteristic (ROC) analysis was conducted via the “timeROC” (Heagerty et al., 2000) package. The AUC was worked out, we considered that MPBs showed good performance for prognosis prediction when the AUC was more than 0.70 (the same as we set in ROC analysis before).

### MPB Mutations and Copy Number Variations

With the aim of screening out mutations and CNVs of genes with high accuracy of predicting OS, all the ACCs and their CNV data from the TCGA database were obtained. The genetic alterations of these genes were screened via the CBio Cancer Genomics Portal (http://www.cbioportal.org/). The correlation between CNVs and relative MPB expression was subsequently identified. The results were measured by ANOVA or Kruskal–Wallis methods. In addition, the relationship between mutations or CNVs of prognostic biomarkers and ACC patients’ survival was screened via survival analysis.

### Functional Exploration of MPBs

Gene set enrichment analysis (GSEA) might help researchers to comprehend the role of genes in biological behaviors. Therefore, we conducted GSEA for MPBs. A total of 79 ACCs were divided into a high-expression group (*n* = 39) and low-expression group (*n* = 40) according to the prognostic biomarkers’ expression median. “c2.cp.kegg.v7.0.symbols.gmt” was chosen as the annotated gene set. We thought a biological pathway of nominal *p* < 0.05, |ES| > 0.6, gene size (n) ≥100, and FDR <25% to be significant. In addition, “DSigDBv1.0.gmt” was downloaded from the Drug SIGnatures DataBase (Yoo et al., 2015) (http://tanlab.ucdenver.edu/DSigDB/DSigDBv1.0/download.html) to explore drugs highly associated with prognostic biomarkers. Also, we set the same cut-off criteria as KEGG pathways identification.

### Exploring the Relationship Between MPBs and Immune Microenvironment

In this part, the association between MPBs and immunocytes was explored via TIMER (Li et al., 2017) (https://cistrome.shinyapps.io/timer/). We thought an MPB with |correlation coefficient (cor) | ≥0.2 and *p* value < 0.05 strongly related to an immune cell infiltrating level as previously found. Furthermore, we explored MPB expressions in 33 different cancer types by using the gene module in TIMER.

### Exploring the Difference of Immune Infiltration Levels Between a Low Expression and High Expression of MPBs

Based on TCGA-ACC data, ESTIMATE scores, immune scores, and stromal scores were firstly evaluated via applying the ESTIMATE algorithm based on R package “estimate” (Yoshihara et al., 2013). Then we divided ACCs into a high- (ESTIMATE, immune, stromal) score group and low- (ESTIMATE, immune, stromal) score group to perform survival analysis via R package “survival”. Moreover, we conducted an unpaired *t* test to test the difference of score levels between a low expression and high expression of MPBs.

### Cox Proportional Hazards Regression Analysis

With the aim of the prognostic value of MPB validation, MPBs and other essential clinical features (gender, age, stage, and laterality) from TCGA-ACC data were selected for OS and DFS univariable Cox analysis. A factor of *p* value < 0.05 was identified and further selected to conduct multivariate Cox analysis. This analysis could determine whether an MPB was independent from the rest of the clinical factors for predicting OS or DFS of ACCs.

### Nomogram Construction

Moreover, with the aim of exploring a simple, quick, and visualized method to predict the possibility of OS or DFS of patients with ACC, two nomograms were constructed (one for OS, the other for DFS) via TCGA-ACC data by using package “rms” (Yizhou et al., 2013). Factors showed meaningful *p* value in Cox regression analysis (including MPBs and clinical features). Calibrate curves were drawn to test the nomogram, the 45° line was defined as the best prediction. In addition, we evaluated the consistency index (C-index) between the actual probability and predicted probability to further measure the prediction effectiveness of the nomogram (Michael and Ralph, 2010). With the aim of avoiding the over-fitting problem, we conducted cross-validation before nomogram construction. Two datasets (GSE10927 and GSE19750) including their OS information were obtained for external verification of the OS-nomogram by calculating C-index and AUC. Meanwhile, GSE76019 and GSE76021 with integral DFS information were included for DFS-nomogram verification.

## Results

### DEG Screening

**FIGURE 1:**
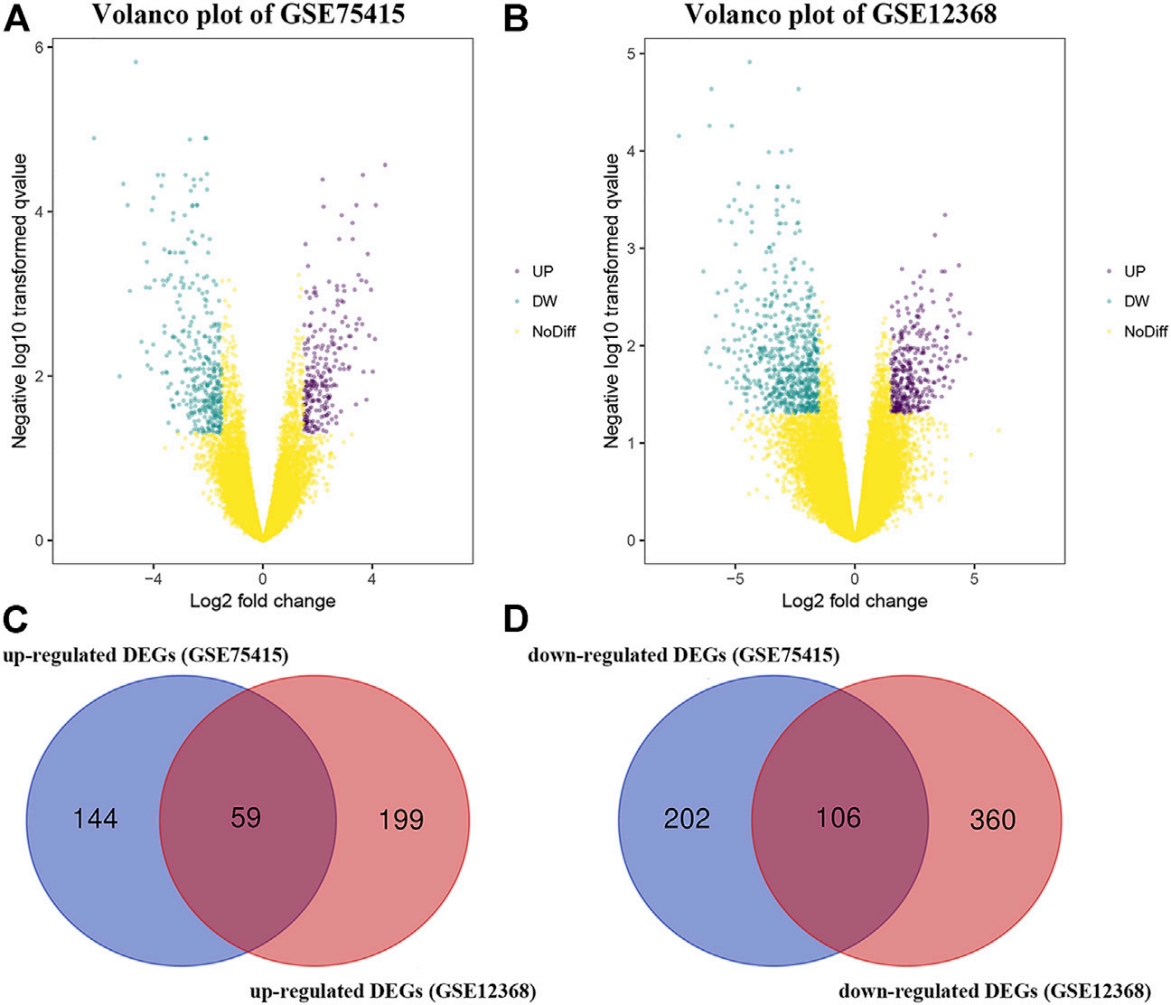
**(A)** Volcano plot visualizing DEGs in GSE75415. **(B)** Volcano plot visualizing DEGs in GSE12368. **(C)** Identification of overlapped upregulated DEGs between GSE75415 and GSE12368. **(D)** Identification of overlapped downregulated DEGs between GSE75415 and GSE12368.

By using the “limma” package in R, we screened 511 DEGs in GSE75415 and 724 DEGs in GSE12368, separately. As shown in Figures 1A,B, 203 over-expressed and 308 low-expressed genes were screened via GSE75415. Furthermore, 258 genes with high expression and 466 genes with low expression were explored via GSE12368. The DEGs both belonged to GSE75415 and GSE12368, including 165 genes (59 upregulated and 106 downregulated) which were finally screened out (Figures 1C,D). All the DEGs we identified are available in Supplementary Table S2.

### Weighted Co-Expression Network Construction and Key Module Identification

**FIGURE 2:**
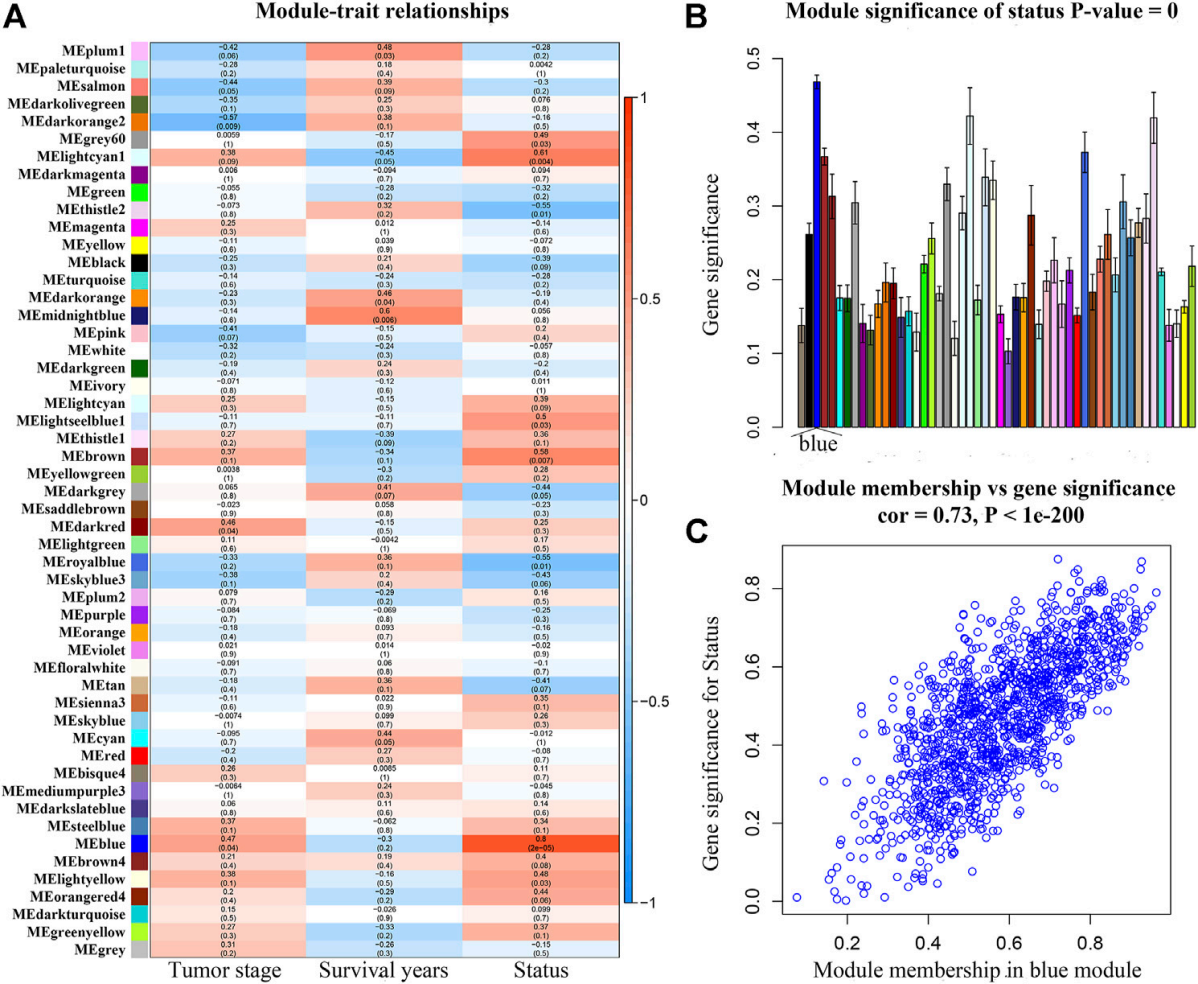
**(A)** Heatmap of the correlation between module eigengenes (MEs) and different clinical information of ACC [tumor stage, survival years (survival time), and survival status]. **(B)** Distribution of average gene significances and errors in the modules associated with the survival status of ACC. **(C)** Scatter plot of module eigengenes related to survival status in the blue module.

After weeding out the outlier samples, a total of 29 samples were used in WGCNA (Supplementary Figures S2A,B). After constructing a co-expression network, the soft-thresholding [beta (β) = 9 (scale free *R* ^2^ = 0.84)] was determined as shown in Supplementary Figures S2C–F. In WGCNA, soft-thresholding was used for further adjacencies evaluation. Immediately, genes were assigned to modules. Also, modules with pairwise correlation of > 0.75 were merged. Finally, 51 modules were screened out (Supplementary Figure S2G). Among them, the most relevant module was the blue module (*P* = 2e-05, *r* = 0.80) (Figure 2A). We also found that the MS of the blue module was the highest compared with the rest of the modules (Figure 2B). As shown in Figure 2C, MM and GS of the blue module showed a significant relationship (*P* = 1e-200, *cor =* 0.73). Thus, we regarded the blue module as the key module in the present study.

### Five Small Molecule Drugs Showed Powerful Potential to Treat ACC

By performing CMap analysis, we could recommend some drugs to treat ACC. As shown in Supplementary Table S3, we screened out eight molecule drugs. Five small molecule drugs including chlorpromazine, trifluoperazine, alpha-estradiol, 15-delta prostaglandin J2, and vorinostat might be potential drugs to treat ACC. The detailed information of the five drugs is shown in Supplementary Table S3.

### Candidate Hub Gene and Hub Gene Identification

Firstly, a PPI network of the 165 DEGs was built. We regarded 74 genes as hub biomarkers because of their high degrees of connectivity (degree ≥ 4, Supplementary Figure S3A). A total of 123 genes with |cor.geneModuleMembership| > 0.8; |cor.geneTraitSignificance| > 0.2 were screened, 99 of which were subsequently chosen via PPI network construction (degree ≥ 4, Supplementary Figure S3B). Finally, 29 genes overlapping between hub genes in DEGs (*n* = 74) and hub genes in the hub modules (*n* = 99) were identified, which were considered to be candidate hub genes.

**FIGURE 3:**
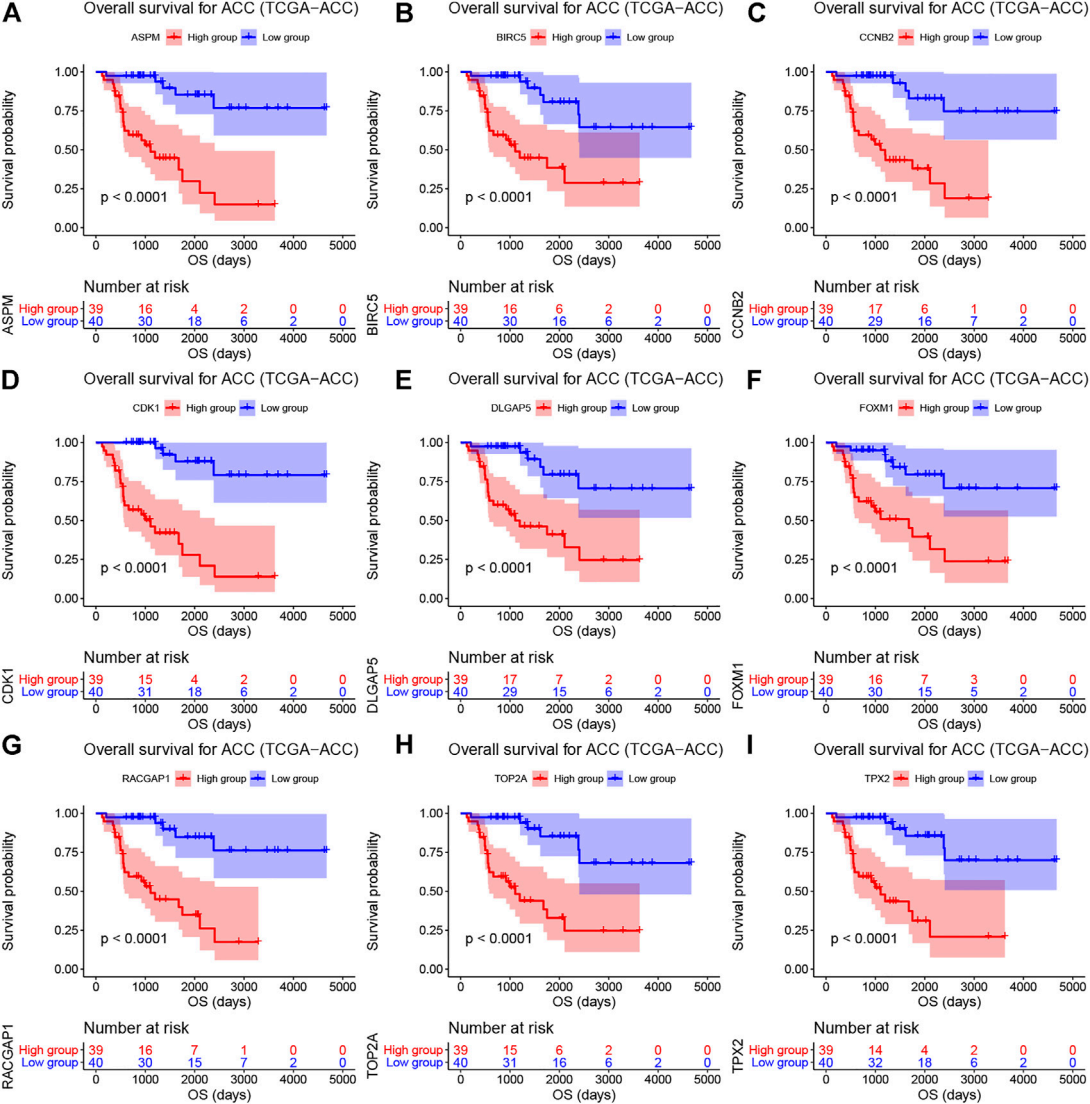
Overall survival analyses on hub genes (ASPM (A), BIRC5 **(B)**, CCNB2 **(C)**, CDK1 **(D)**, DLGAP5 **(E)**, FOXM1 **(F)**, RACGAP1 **(G)**, TOP2A **(H)**, and TPX2 **(I)**) based on TCGA-ACC data. Survival curves for patients in different groups. Red lines represent high expression of hub genes, while blue lines represent low expression of hub genes.

As shown in Supplementary Table S4, the survival analysis indicated that 24 genes were associated with overall survival (OS) and diseases-free survival (DFS) in TCGA-ACC data. A total of 19 genes were associated with OS in GSE19750. Overall, 21 genes in GSE76019 and 29 genes in GSE76021 were associated with event-free survival (EFS). Genes that showed a significant *p* value (*p* < 0.05) in these survival analyses were considered to be hub genes related to survival and prognosis of patients with ACC. Finally, nine genes [ASPM (abnormal spindle microtubule assembly), BIRC5 (baculoviral IAP repeat containing 5), CCNB2 (cyclin B2), CDK1 (cyclin dependent kinase 1), DLGAP5 (DLG associated protein 5), FOXM1 (forkhead box M1), RACGAP1 (Rac GTPase activating protein 1), TOP2A (DNA topoisomerase II alpha), and TPX2 (TPX2 microtubule nucleation factor)] were screened out. The results of survival analyses of the hub genes are shown in Figure 3 (OS, TCGA-ACC data), Supplementary Figure S4 (DFS, TCGA-ACC data), Supplementary Figure S5 (OS, GSE19750), Supplementary Figure S6 (EFS, GSE76019), and Supplementary Figure S7 (EFS, GSE76021). Also, we explored univariate Cox analysis for the nine genes based on TCGA-ACC data, GSE76019, and GSE76021. As shown in Supplementary Table S5, the result was consistent with what we got for survival analysis.

### Hub Gene Validation

Based on GSE10927, GSE19750, GSE75415, GSE76019, GSE76021, and TCGA-ACC data, the stage plots of hub genes were determined and these genes did not perform as well as we expected. In TCGA-ACC data, ASPM (F = 6.939, *p* = 0.001), BIRC5 (F = 3.368, *p* = 0.034), CCNB2 (F = 4.844, *p* = 0.009), CDK1 (F = 6.779, *p* = 0.001), DLGAP5 (F = 4.170, *p* = 0.014), FOXM1 (F = 7.569, *p* = 0.001), RACGAP1 (F = 4.717, *p* = 0.009), TOP2A (F = 4.687, *p* = 0.008), and TPX2 (F = 5.232, *p* = 0.005) were significantly associated with tumor stage (Supplementary Table S6). In GSE10927, only ASPM showed a significant *p* value (F = 4.254, *p* = 0.030) (Supplementary Table S6). In GSE19750, GSE75415, and GSE76019, unfortunately none of these hub genes were closely relevant to tumor stage (Supplementary Table S6). In GSE76021, only CCNB2 (F = 7.569, *p* = 0.001) was significantly related to tumor stage (Supplementary Table S6). As for grade plots, the results of the unpaired *t* test suggested that ASPM, BIRC5, CCNB2, CDK1, DLGAP5, FOXM1, RACGAP1, TOP2A, and TPX2 were closely related to tumor grade based on GSE10927 (the *p* values are shown in Supplementary Table S7). In GSE19750, only CCNB2 (F = 6.271, *p* = 0.013) was significantly associated with tumor grade (Supplementary Table S6). In bioinformatics analysis of each dataset (GSE10927, GSE12368, GSE19750, and GSE75415), all the hub genes were highly expressed in ACCs compared to normal tissue (Supplementary Table S8).

### ROC and DCA

By using GSE10927, GSE12368, GSE19750, and GSE75415, ROC curve analysis was performed and the AUC was evaluated for distinguishing ACCs and normal samples. The AUC values of hub genes were greater than 0.84, which suggested that all of the hub genes could distinguish ACCs from normal tissues well (Table 1). Also, the AUC was calculated to distinguish localized ACC (stages I or II) and advanced ACC (stages III or IV) based on all the datasets we mentioned in this study. In TCGA-ACC data, all the hub genes could distinguish localized ACC and advanced ACC well (Table 1). In GSE19750, BIRC5 (AUC = 0.727) and TOP2A (AUC = 0.765) worked well (Table 1). In GSE76019, only ASPM (AUC = 0.713) could distinguish localized ACC and advanced ACC well (Table 1). In GSE10927, GSE75415, and GSE76021, none of these hub genes could distinguish localized ACC from advanced ACC well (Table 1), which is not what we expected. According to the results of distinguishing ACC of low grade and ACC of high grade, all these genes showed a significant *p* value (AUC > 0.80) based on GSE10927 (Table 1). But in GSE19750, BIRC5 (AUC = 0.495) could not distinguish ACC of low grade and ACC of high grade well (Table 1). As for results of AUC to distinguish ACA and ACC, the AUC values of hub genes were greater than 0.85 by using GSE10927 and GSE12368, which suggested that all the hub genes worked well (Table 1). But in GSE75415, only FOXM1 (AUC = 0.747), TOP2A (AUC = 0.726), and TPX2 (AUC = 0.726) could distinguish ACC and ACA well. All the results of this part are shown in Table 1. As for the DCA results, eight of the hub genes (ASPM, BIRC5, CDK1, DLGAP5, FOXM1, RACGAP1, TOP2A, and TPX2) expressed a strong potential for clinical practice (Supplementary Figure S8). Whatever the threshold probability (Pt) expressed, the eight genes displayed great potential. For CCNB2, it performed well, only Pt was approximately between 0.20 and 0.60. All in all, these results suggested that though these hub genes performed well in some datasets, they need to be tested by more in-depth study.

**TABLE 1:** AUC of hub genes.

	Normal tissues vs. ACC				Stages I/II vs. stages III/IV						Low grade vs. high grade		ACA vs. ACC		
Genes	GSE10927	GSE12368	GSE19750	GSE75415	GSE10927	GSE19750	GSE75415	GSE76019	GSE76021	TCGA	GSE10927	GSE19750	GSE10927	GSE12368	GSE75415
ASPM	0.982	0.986	0.957	0.917	0.532	0.553	0.513	0.713	0.593	0.751	0.86	0.859	0.972	0.995	0.663
BIRC5	0.968	0.861	0.932	0.895	0.511	0.727	0.563	0.661	0.591	0.762	0.823	0.495	0.979	0.859	0.695
CCNB2	0.991	1	0.852	0.902	0.481	0.606	0.538	0.654	0.598	0.799	0.858	0.901	0.986	1	0.674
CDK1	0.988	0.858	0.966	0.91	0.474	0.58	0.6	0.671	0.529	0.799	0.877	0.82	0.977	0.99	0.674
DLGAP5	0.979	0.931	0.847	0.88	0.489	0.564	0.613	0.654	0.544	0.76	0.912	0.875	0.987	0.885	0.642
FOXM1	0.994	0.917	0.952	0.914	0.57	0.67	0.575	0.63	0.618	0.752	0.813	0.747	0.98	0.938	0.747
RACGAP1	1	0.944	0.989	0.925	0.53	0.561	0.475	0.637	0.544	0.745	0.896	0.823	1	0.979	0.589
TOP2A	0.97	0.858	0.969	0.955	0.538	0.765	0.463	0.682	0.578	0.763	0.827	0.685	0.979	0.901	0.726
TPX2	0.97	1	1	0.962	0.5	0.644	0.488	0.626	0.578	0.733	0.823	0.859	0.968	0.953	0.726

Note: AUC: area under curve.

### Hub Gene-Associated Biological Pathways

GO analysis indicated that candidate hub genes were involved in 10 biological processes (BPs), including nuclear division, organelle fission, mitotic nuclear division, chromosome segregation, nuclear chromosome segregation, sister chromatid segregation, mitotic sister chromatid segregation, cell cycle checkpoint, regulation of chromosome segregation, and microtubule cytoskeleton organization involved in mitosis (Supplementary Figure S9A). As for the KEGG pathways, candidate biomarkers were majorly associated with cell cycle, progesterone-mediated oocyte maturation, oocyte meiosis, cellular senescence, and p53 signaling pathway (Supplementary Figure S9B). To summarize, we found that candidate hub genes were majorly associated with cell cycle and DNA replication-related biological pathways.

### Four MPBs Showed Powerful Potential to Predict OS

**FIGURE 4:**
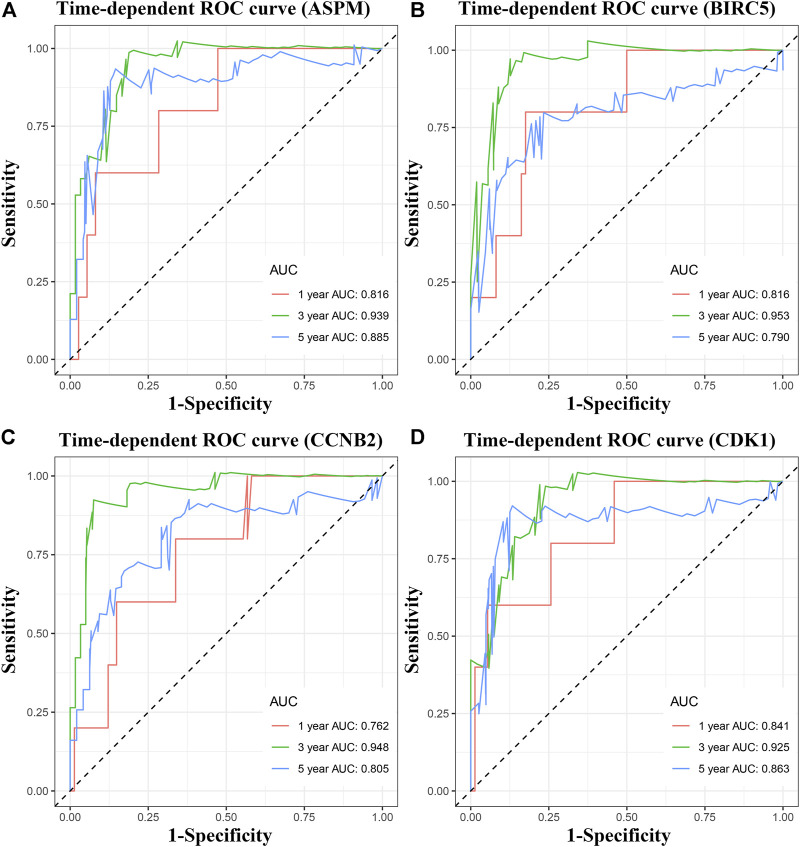
Time dependent ROC analyses at 1, 3, and 5 years based on MPB expression. **(A)** ASPM, **(B)** BIRC5, **(C)** CCNB2, and **(D)** CDK1.

To pick out some genes with great value for predicting OS among the nine hub genes, three methods including LDA, KNN, and SVM from the machine learning field were included in this part. As Table 2 describes, though all the sixteen biomarkers might perform well in recognizing ACCs from alive ACC samples (the average accuracy ≥ 0.70), four MPBs including ASPM (average accuracy = 0.8228), BIRC5 (average accuracy = 0.8059), CCNB2 (average accuracy = 0.8080), and CDK1 (average accuracy = 0.8080) were screened out for more accurate prediction of OS. Furthermore, time-dependent ROC analysis for the four genes was conducted. We concluded that all the four MPBs could predict OS of patients with ACC well (Figure 4). For ASPM, the AUCs of 1-, 3-, and 5-years OS were 0.816, 0.939, and 0.885, respectively (Figure 4A). For BIRC5, the AUCs of 1-, 3-, and 5-years OS were 0.816, 0.953, and 0.790, respectively (Figure 4B). For CCNB2, the AUCs of 1-, 3-, and 5-years OS were 0.762, 0.948, and 0.805, respectively (Figure 4C). For CDK1, the AUCs of 1-, 3-, and 5-years OS were 0.841, 0.925, and 0.863, respectively (Figure 4D).

**TABLE 2:** The accuracy of classification of LDA-based classifier, KNN-based classifier, linear-SVM-based classifier, polynomial-SVM-based classifier, RBF-SVM-based classifier, and sigmoid-kernel-SVM based classifier.

TCGA-ACC	LDA	KNN	Linear-SVM	Polynomial-SVM	RBF-SVM	Sigmoid-kernel SVM	Average accuracy
ASPM	0.8354	0.8101	0.8101	0.8228	0.8354	0.8228	0.8228
BIRC5	0.8101	0.8228	0.8101	0.7722	0.8101	0.8101	0.8059
CCNB2	0.7975	0.8481	0.7975	0.8101	0.7975	0.7975	0.8080
CDK1	0.7848	0.8354	0.8101	0.8101	0.8101	0.7975	0.8080
DLGAP5	0.7595	0.7975	0.7595	0.7722	0.7722	0.7848	0.7743
FOXM1	0.7595	0.7848	0.7595	0.7595	0.7468	0.7722	0.7637
RACGAP1	0.7975	0.7848	0.7975	0.7975	0.7975	0.6329	0.7680
TOP2A	0.7848	0.7975	0.7342	0.7975	0.7975	0.7089	0.7701
TPX2	0.7342	0.7848	0.7342	0.7595	0.7468	0.7342	0.7490

Note: LDA: linear discriminant analysis; KNN: K-nearest neighbor; RBF: radial basis function; SVM: support vector machine.

### Mutations and CNVs of MPBs Were Associated With OS of Patients With ACC

**FIGURE 5:**
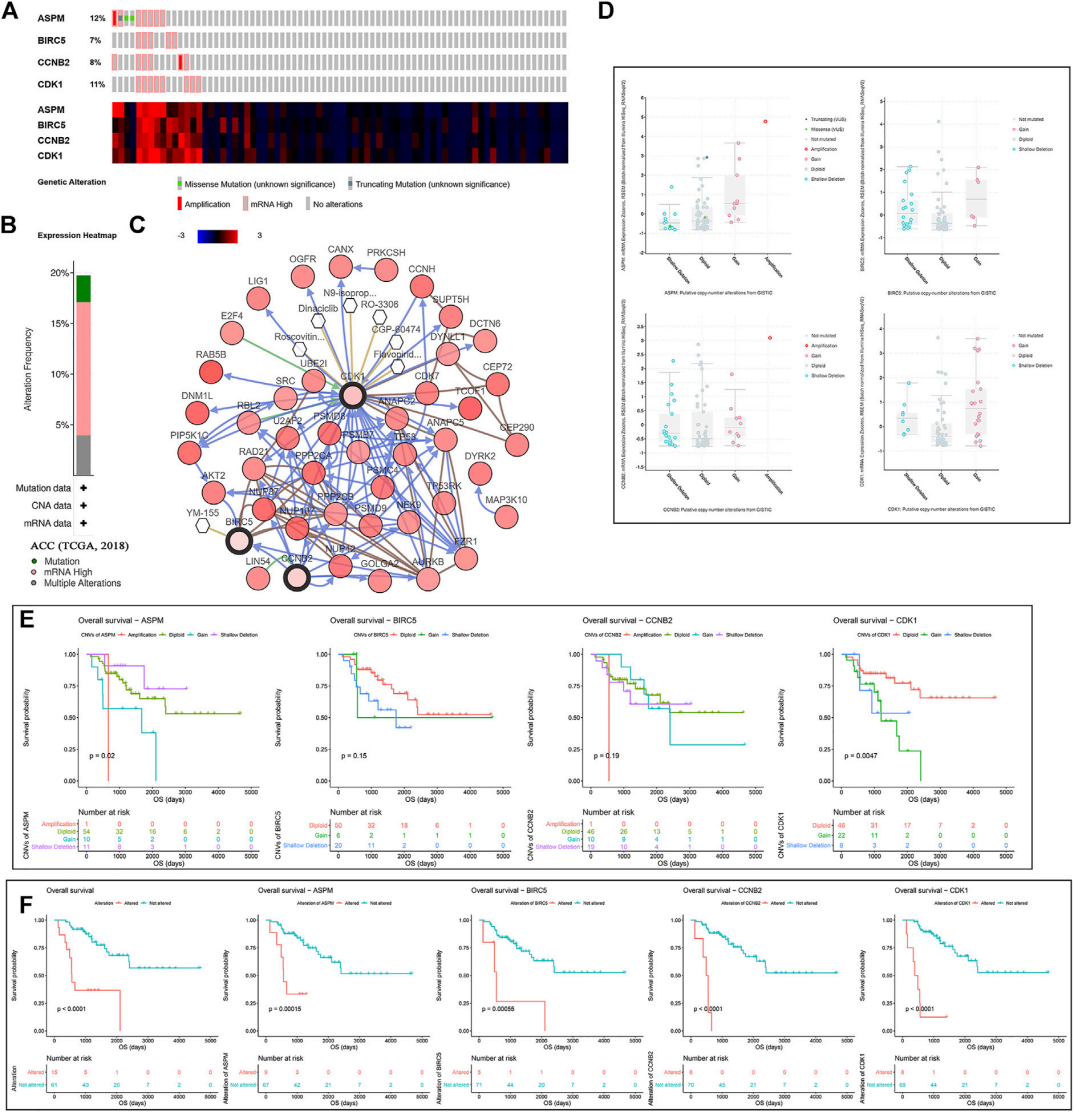
A summary of mutations and CNVs of MPBs. **(A)** Genetic alterations associated with MPBs and expression heatmap of MPBs based on the data from TCGA. **(B)** The total alteration frequency of MPBs in TCGA-ACC is illustrated. **(C)** The network contains 54 nodes, including our 4 query genes and the 50 most frequently altered neighbor genes. The relationship between hub genes and tumor drugs is also illustrated. **(D)** Correlation between different CNV patterns and mRNA expression levels of MPBs respectively. **(E)** Survival analysis of ACC patients with CNVs of MPBs based on TCGA ACC data. **(F)** Survival analysis of ACC patients with mutations of MPBs based on TCGA ACC data.

According to the result, four MPBs were altered in 15 (20%) of 76 ACC patients (Figure 5B). The most altered gene was ASPM (12%, Figure 5A). And we further concluded that mRNA high was the main type (Figure 5A). For exploring the relationship between gene expression and gene alteration, we found that genes with more alterations were more likely to be highly expressed. Figure 5C shows the network containing 54 nodes (including 4 MPBs and 50 most altered neighbor genes). In addition, this network also demonstrated that CDK1 and BIRC5 were the targets of some kinds of anticancer drugs, which suggested that ASPM and CCNB2 might be new therapeutic targets to treat ACC. Moreover, CNVs of ASPM (gains), BIRC5 (shallow deletions, gains), CCNB2 (shallow deletions, gains), and CDK1 (shallow deletions, gains) caused their higher expressions compared with samples without CNVs (diploids), which demonstrated that CNVs of MPBs were associated with their expression levels (Figure 5D).

As for the effect of CNVs and mutations of genes on OS, we concluded that ACCs with ASPM shallow deletions (*p* = 0.0200) had better OS compared to those affected by ASPM copy number gains. In addition, there was a contrary conclusion that ACCs with shallow deletions in CDK1 (*p* = 0.0047) had poor OS (Figure 5E). Moreover, ACCs of alterations in the four biomarkers had worse OS (total alterations: *p* < 0.0001; ASPM alterations: *p* = 0.00015; BIRC5 alterations: *p* = 0.00055; CCNB2 alterations: *p* < 0.0001; CDK1 alterations: *p* < 0.0001; Figure 5F).

### Identification of MPB-Related Biological Pathways and Drugs

With the standards set before, interestingly, ASPM, BIRC5, CCNB2, and CDK1 were involved in just one KEGG signaling pathway called cell cycle as shown in Supplementary Table S8 (*p* values are also described in Supplementary Table S8). As for the enriched drugs, ASPM was significantly enriched in 6 drugs, BIRC5 was associated with 6 drugs, CCNB2 was related to 11 drugs, and CDK1 was enriched in 6 drugs (Table 3).

**TABLE 3:** Gene set enrichment analyses in four hub genes’ (ASPM, BIRC5, CCNB2, CDK1) high-expression phenotype.

Gene symbol	Reference gene set	Name	Size	ES	NES	NOM *p*-val	FDR *q*-val
ASPM	c2.cp.kegg.v7.0.symbols.gmt	KEGG_CELL_CYCLE	122	−0.7048	−2.1129	0.0000	0.0029
	DSigDBv1.0.gmt	LUCANTHONE_CTD_00006227	202	−0.8190	−1.8734	0.0000	0.0357
		MONOBENZONE_PC3_DOWN	196	−0.6530	−2.1874	0.0000	0.0160
		8-AZAGUANINE_PC3_DOWN	192	−0.6831	−2.1539	0.0000	0.0160
		THIOGUANOSINE_MCF7_DOWN	145	−0.6482	−2.0313	0.0000	0.0392
		AZACYCLONOL_MCF7_UP	123	−0.6266	−1.5119	0.0481	0.2097
		RESVERATROL_MCF7_DOWN	100	−0.8126	−1.8743	0.0000	0.0377
BIRC5	c2.cp.kegg.v7.0.symbols.gmt	KEGG_CELL_CYCLE	122	−0.7182	−2.2001	0.0000	0.0000
	DSigDBv1.0.gmt	DASATINIB_CTD_00004330	474	−0.6359	−1.7919	0.0000	0.0585
		LUCANTHONE_CTD_00006227	202	−0.8058	−1.8166	0.0000	0.0538
		MONOBENZONE_PC3_DOWN	196	−0.6272	−2.0778	0.0020	0.0415
		8-AZAGUANINE_PC3_DOWN	192	−0.6720	−2.1018	0.0000	0.0488
		THIOGUANOSINE_MCF7_DOWN	145	−0.6460	−1.9901	0.0000	0.0248
		RESVERATROL_MCF7_DOWN	100	−0.8303	−1.9074	0.0000	0.0322
CCNB2	c2.cp.kegg.v7.0.symbols.gmt	KEGG_CELL_CYCLE	122	−0.6765	−2.0239	0.0000	0.0110
	DSigDBv1.0.gmt	DASATINIB_CTD_00004330	474	−0.6032	−1.7238	0.0041	0.0930
		LUCANTHONE_CTD_00006227	202	−0.7869	−1.7530	0.0000	0.0810
		MONOBENZONE_PC3_DOWN	196	−0.6328	−2.0912	0.0000	0.0598
		8-AZAGUANINE_PC3_DOWN	192	−0.6633	−1.9833	0.0000	0.0525
		THIOGUANOSINE_MCF7_DOWN	145	−0.6569	−1.9812	0.0000	0.0481
		PRENYLAMINE_MCF7_UP	140	−0.6343	−1.6213	0.0120	0.1535
		MEFLOQUINE_MCF7_UP	136	−0.6014	−1.5385	0.0373	0.2092
		AZACYCLONOL_MCF7_UP	123	−0.6562	−1.5766	0.0237	0.1748
		AZACITIDINE_PC3_UP	115	−0.6318	−1.4942	0.0339	0.2350
		FENDILINE_MCF7_UP	112	−0.6230	−1.5397	0.0354	0.2081
		RESVERATROL_MCF7_DOWN	100	−0.8189	−1.8592	0.0000	0.0580
CDK1	c2.cp.kegg.v7.0.symbols.gmt	KEGG_CELL_CYCLE	122	−0.7160	−2.1710	0.0000	0.0049
	DSigDBv1.0.gmt	DASATINIB_CTD_00004330	474	−0.6174	−1.7945	0.0020	0.0569
		LUCANTHONE_CTD_00006227	202	−0.8038	−1.8426	0.0000	0.0481
		MONOBENZONE_PC3_DOWN	196	−0.6407	−2.1553	0.0000	0.0338
		8-AZAGUANINE_PC3_DOWN	192	−0.6686	−2.0766	0.0000	0.0501
		THIOGUANOSINE_MCF7_DOWN	145	−0.6511	−2.0313	0.0000	0.0485
		RESVERATROL_MCF7_DOWN	100	−0.8223	−1.8556	0.0000	0.0474

Note: ES, enrichment score; NES, normalized enrichment score; NOM *p*-val, nominal *p* value; FDR, false discovery rate q value.

### MPB Expressions Were Related to Immune Infiltration Level in ACC

Immune infiltration level was an independent predictor of sentinel lymph node status and survival in tumors. Here we assessed the correlation of MPB expressions with immune infiltration level in ACC. The analysis concluded that ASPM expression was positively relevant to tumor purity (cor = 0.300, *p* = 0.009) and infiltrating levels of B cells (cor = 0.272, *p* = 0.020) and dendritic cells (cor = 0.236, *p* = 0.044) but had no significant correlations with infiltrating levels of CD8 + T cells, CD4 + cells, macrophages, and neutrophils (Supplementary Figure S10A). Unfortunately, BIRC5 expression was not related to tumor purity or infiltrating levels of immune cells (Supplementary Figure S10B). Moreover, there was a positive relationship between CCNB2 expression and tumor purity (cor = 0.268, *p* = 0.021) and infiltrating level of dendritic cells (cor = 0.238, *p* = 0.043) (Supplementary Figure S10C). As for CDK1, the expression of CDK1 only had a positive correlation with tumor purity (cor = 0.283, *p* = 0.015) (Supplementary Figure S10D). To summarize, we found that ASPM, CCNB2, and CDK1 expressions were significantly associated with tumor purity, which could be a sign that ASPM, CCNB2, and CDK1 played specific roles in immune infiltration in ACC.

In addition, as shown in Supplementary Figure S13, ASPM expression (Supplementary Figure S11A), BIRC5 expression (Supplementary Figure S11B), and CCNB2 expression (Supplementary Figure S11C) were significantly higher in 17 types of cancer compared with adjacent normal tissues. In 25 cancer types, CDK1 showed an upregulated trend when comparing to normal tissues (Supplementary Figure S11D). Unfortunately, there was a lack of adjacent normal tissues in ACC (based on TCGA data), and we could not compare the expressions between ACC and normal tissue of MPBs. But as per our previous results in this study, all the MPBs were over-expressed (based on GSE10927, GSE12368, GSE19750, and GSE75415).

### Association of MPB Expressions With Immune Microenvironment Score Levels

**FIGURE 6:**
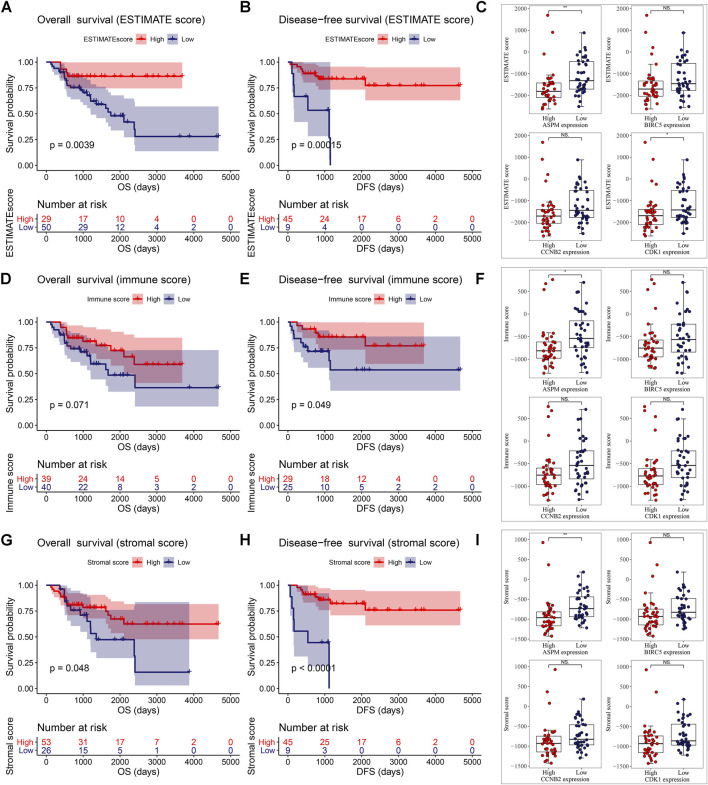
ESTIMATE scores were associated with overall survival **(A)** and disease-free survival **(B)** of patients with ACC. Correlation of MPB **(C)** expression with ESTIMATE scores in ACC. Immune scores were associated with overall survival **(D)** and disease-free survival **(E)** of patients with ACC. Correlation of MPB **(F)** expression with immune scores in ACC. Stromal scores were associated with overall survival **(G)** and disease-free survival **(H)** of patients with ACC. Correlation of MPB **(I)** expression with stromal scores in ACC. *: *p* < 0.05; **: *p* < 0.01; NS: no significance.

Then we found that patients with ACC in the high ESTIMATE-score group had better OS and disease-free survival (DFS). Patients of low ASPM expression and low CDK1 expression had higher ESTIMATE scores (Figure 6C). As shown in Figure 6D, there was a trend where ACCs which had a high-immune score had superior OS compared to those with a low-immune score. Meanwhile, patients with a high-immune score had better disease-free survival (DFS) compared with patients with a low-immune score, significantly (Figure 6E). The unpaired *t* test indicated that there was a negative relationship between ASPM expression and immune score level (Figure 6F). As shown in Figure 6G, an ACC patient with a high-stromal score had better OS. Meanwhile, patients with a high-stromal score had better disease-free survival (DFS) compared with patients with a low-stromal score, significantly (Figure 6H). The unpaired *t* test also suggested that there was a negative relationship between ASPM expression and stromal score level (Figure 6I). These results demonstrated that high expressions of MPBs had worse OS and DFS in ACC patients indirectly.

### Prognostic Value of the Four Biomarkers

According to the result of univariate Cox analysis (Table 4), ASPM, BIRC5, CCNB2, CDK1, and pathologic stage were interfering factors of OS. *p* values are shown in Table 4. Subsequent multivariate Cox analysis confirmed that ASPM could predict the prognosis of ACC patients by individual. By using the Coxph function in R package “survival”, we conducted a Schoenfeld individual test for investigating the proportional hazards assumption. The global Schoenfeld test showed no significance (*p* = 0.1936, Supplementary Figure S12A). Also, each variable including age (*p* = 0.6709), gender (*p* = 0.6919), laterality (*p* = 0.6219), pathologic stage (*p* = 0.1688), ASPM (*p* = 0.3394), BIRC5 (*p* = 0.1285), CCNB2 (*p* = 0.4813), and CDK1 (*p* = 0.0657) was not statistically significant (*p* > 0.05, Supplementary Figure S12A). Thus, this Cox model conformed to the proportional hazards assumption. For DFS, ASPM (hazard ratio = 2.768, 95% CI of ratio: 1.450–5.284, *p* = 0.002), CCNB2 (hazard ratio = 2.441, 95% CI of ratio: 1.422–4.189, *p* = 0.001), CDK1 (hazard ratio = 1.928, 95% CI of ratio: 1.040–3.576, *p* = 0.037), and pathologic stage (hazard ratio = 1.848 95% CI of ratio: 1.024–3.338, *p* = 0.042) were interfering factors of DFS via univariate Cox analysis. ASPM must be the most important factor for DFS of ACC patients suggested by multivariate Cox analysis (hazard ratio = 7.335, *p* = 0.012). By using the Coxph function in R package “survival”, we conducted a Schoenfeld individual test for investigating the proportional hazards assumption. The global Schoenfeld test showed no significance (*p* = 0.8934, Supplementary Figure S12B). Also, each variable including age (*p* = 0.3864), gender (*p* = 0.8702), laterality (*p* = 0.5409), pathologic stage (*p* = 0.5914), ASPM (*p* = 0.3790), BIRC5 (*p* = 0.4194), CCNB2 (*p* = 0.5385), and CDK1 (*p* = 0.5581) was not statistically significant (*p* > 0.05, Supplementary Figure S12B). Thus, this Cox model conformed to the proportional hazards assumption.

**TABLE 4:** Cox univariable and multivariable analyses of overall survival (OS) and disease-free survival (DFS).

	Variable	Univariate analysis				Multivariate analysis			
		HR	LCI	UCI	*p* value	HR	LCI	UCI	*p* value
Overall survival (OS)	ASPM	3.184	2.157	4.701	<0.001	3.262	1.138	9.350	0.028
	BIRC5	2.862	1.946	4.209	<0.001	1.941	0.811	4.646	0.137
	CCNB2	2.812	1.935	4.085	<0.001	0.989	0.443	2.208	0.978
	CDK1	3.873	2.342	6.405	<0.001	0.489	0.140	1.710	0.263
	Age	1.011	0.987	1.036	0.365				
	Gender	1.000	0.468	2.135	0.999				
	Laterality	0.841	0.394	1.796	0.654				
	Pathologic stage	2.912	1.858	4.562	<0.001	1.943	1.191	3.170	0.008
Disease-free survival (DFS)	ASPM	2.768	1.450	5.284	0.002	7.335	1.538	34.994	0.012
	BIRC5	1.674	0.979	2.865	0.060				
	CCNB2	2.441	1.422	4.189	0.001	2.297	0.717	7.362	0.162
	CDK1	1.928	1.040	3.576	0.037	0.114	0.021	0.617	0.012
	Age	0.998	0.963	1.034	0.919				
	Gender	2.386	0.665	8.563	0.182				
	Laterality	0.423	0.132	1.348	0.146				
	Pathologic stage	1.848	1.024	3.338	0.042	1.205	0.597	2.433	0.602

### Clinical Application of MPBs

**FIGURE 7:**
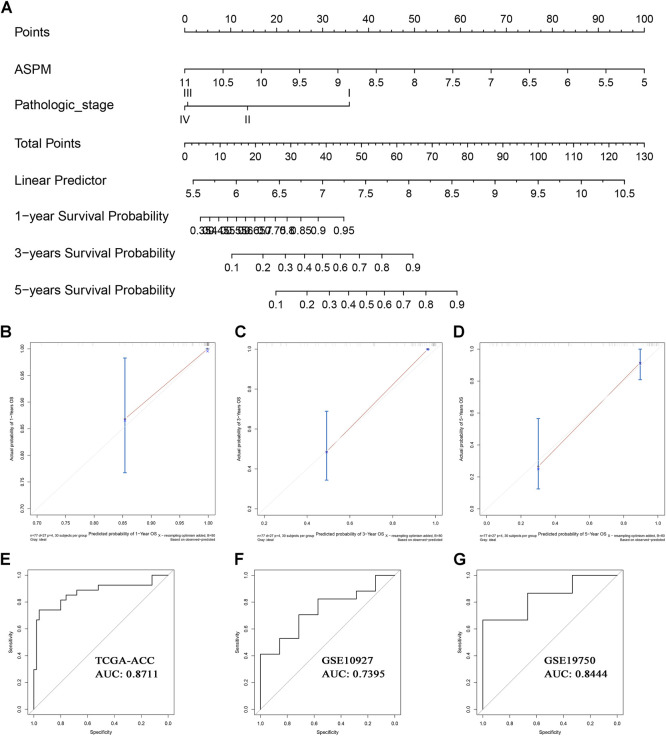
The nomogram for predicting the proportion of ACC patients with 1-, 3-, or 5-years OS **(A)**. The calibration plots for predicting 1- **(B)**, 3- **(C)**, or 5- **(D)** year OS. Receiver operating characteristic (ROC) curves and area under the curve (AUC) statistics to evaluate the diagnostic efficiency of the nomogram in TCGA-ACC data **(E)**, GSE10927 **(F)**, and GSE19750 **(G)**.

**FIGURE 8:**
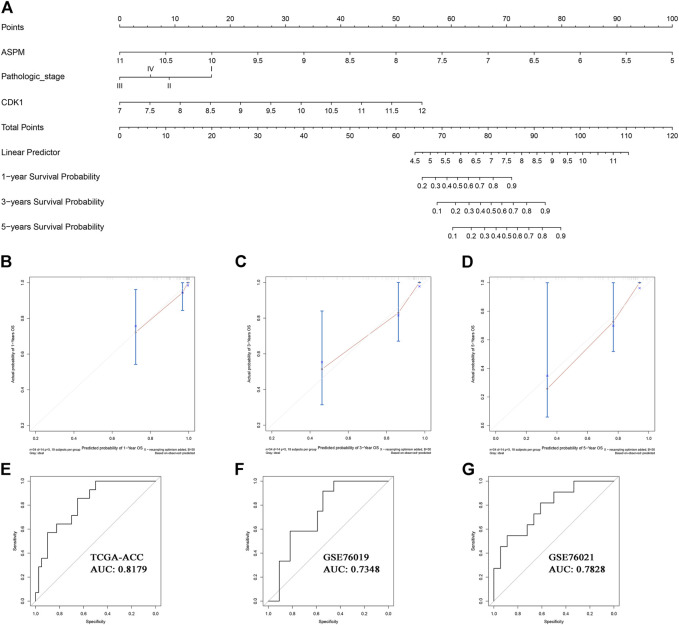
The nomogram for predicting the proportion of ACC patients with 1-, 3-, or 5-years DFS **(A)**. The calibration plots for predicting 1- **(B)**, 3- **(C)**, or 5- **(D)** year DFS. Receiver operating characteristic (ROC) curves and area under the curve (AUC) statistics to evaluate the diagnostic efficiency of the nomogram in TCGA-ACC data **(E)**, GSE76019 **(F)**, and GSE76021**(G)**.

Based on the factors which showed a significant *p* value in multivariate Cox analysis, we constructed two nomograms (one for OS, the other for DFS) to make better use of these prognostic biomarkers. Two features including ASPM and pathologic stage were used for construction of the OS-nomogram (Figure 7A) meanwhile the DFS-nomogram contained three factors including ASPM, CDK1, and pathologic stage (Figure 8A). By reviewing the C-index and AUC, we found that both the two nomograms performed well in survival prediction. The OS-nomogram could make accurate predictions about ACC patients’ OS via TGCA-ACC data (C-index: 0.875; AUC: 0.871; Figure 7E), GSE10927 (C-index: 0.748; AUC: 0.740; Figure 7F), and GSE19750 (C-index: 0.612; AUC: 0.844; Figure 7G). As for the predication performance of the DFS-nomogram, it was obvious that the DFS-nomogram showed accurate prediction potential of ACC patients’ DFS based on TCGA-ACC data (C-index: 0.834; AUC: 0.818; Figure 8E), GSE76019 (C-index: 0.694; AUC: 0.735; Figure 8F), and GSE76021 (C-index: 0.749; AUC: 0.783; Figure 8G). As the result of the calibration curve suggested, both the OS-nomogram (Figures 7B–D) and DFS-nomogram (Figures 8B–D) had good prediction effectiveness compared to the ideal model for a nomogram’s 1-, 3-, and 5-years OS estimates.

## Discussion

Though ACC is a relatively orphan malignant tumor, most ACCs are diagnosed in advanced stages (Guillaume et al., 2014). The 5-years survival rate of ACC is still not satisfactory (only 35% as reported) (Guillaume et al., 2014). In consideration of the poor prognosis of ACC patients, it was of urgent need to explore a few effective and novel biomarkers predicting the survival and prognosis of patients with ACC by integrative bioinformatics analysis. Moreover, we attempted to provide clinicians a simple, quick, and accurate method for survival prediction by constructing nomograms.

Based on WGCNA, DEG, and PPI analysis, we identified nine genes which might be candidate biomarkers in ACC. We further explored the potential functions of these hub genes. The results of functional enrichment analysis suggested that the hub genes were majorly enriched in cell cycle and DNA replication-related pathways. Cell cycle is the basic process of cell proliferation (Kaistha et al., 2015). Interestingly, two previous studies demonstrated that most of the nine biomarkers were effectively involved in the cell cycle of renal cell carcinoma (Chen et al., 2018; Wang et al., 2018), which made us more confident in our findings. Yuan at al. confirmed that ASPM, FOXM1, RACGAP1, and TPX2 were significantly associated with not only tumor progression but also prognosis of ACC (Yuan et al., 2018). In the same datasets they used (TCGA-ACC data and GSE19750), we came to the same conclusion. But in other datasets (GSE10927, GSE19750, GSE75415, GSE76019, and GSE76021), these genes were not significantly related to tumor progression as we expected. Therefore, we thought there needs to be stronger evidence and more in-depth validation for exploring the correlation between the nine genes and tumor progression. Previous studies indicated that DNA replication regulation was one of the core events of cell cycle regulation (Fragkos et al., 2015). Cell cycle and DNA replication influenced each other and there existed a complicated relationship between them (Lin et al., 2017). To summarize, the conclusions of the above studies provided strong support for suggesting the sixteen genes as new prognostic biomarkers for ACC patients.

Then four MPBs (including ASPM, BIRC5, CCNB2, and CDK1) with higher accuracy in predicting survival were screened out among the nine genes by performing LDA, KNN, SVM, and time-dependent ROC. The effect of mutations and CNVs of MPBs were subsequently evaluated. These MPBs were altered in 15 (20%) patients with ACC. ASPM was altered most and mRNA high was the main type. The next-step process concluded that mutations and CNVs of MPBs were related to ACC patients’ OS.

Considering that the tumor immune microenvironment showed a strong correlation with progression and treatment of tumors. We also attempted to explore the relationship in this study. The results suggested that MPB expressions were significantly correlated with immune infiltration level in ACC. Moreover, high expressions of MPBs were effectively associated with worse survival in patients with ACC.

In addition, the CMap analysis demonstrated that five small molecule drugs including chlorpromazine, trifluoperazine, alpha-estradiol, 15-delta prostaglandin J2, and vorinostat might be novel drugs for ACC treatment. These MPBs were also significantly enriched in cell cycle. As for the enriched drugs, ASPM was significantly enriched in 6 drugs, BIRC5 was associated with 6 drugs, CCNB2 was related to 11 drugs, and CDK1 was enriched in 6 drugs. All in all, these drugs might be potential choices for treating ACC.

A nomogram mainly assigns scores to each value level of each influencing factor through the contribution of each influencing factor to the outcome variable in the model, and then adds each score to obtain the total score. Finally, through the functional conversion relationship between the total score and the occurrence probability of the outcome event, the predicted value of the individual outcome event is calculated. In this manuscript, based on the factors which showed a significant *p* value in multivariate Cox analysis, we constructed two nomograms (one for OS, the other for DFS) to make better use of these prognostic biomarkers. Clinicians might realize the individualized and accurate prediction of ACC patients via the two nomograms.

We also have to discuss the deficiencies of our study. Firstly, there was a lack of validation by using *in vitro* or *in vivo* models. Therefore, we will verify the four genes by conducting histology or animal experiments in further research. Secondly, although we identified and validated the four MPBs which were related to prognosis of ACC patients by using several independent datasets, these datasets were of small size, and there was a lack of clinical trials by using samples from patients. Therefore, we need to verify our results by collecting large amounts of patient samples and relevant clinical data in a further study.

In conclusion, we performed eight independent methods to screen nine hub genes related to survival and prognosis of ACC by using seven independent datasets. Four MPBs among them were further screened out, which performed well in ACC survival and prognosis prediction. Furthermore, two nomograms including the OS-nomogram and DFS-nomogram were established, which provided clinicians with a quick, accurate, and visualized method for OS and DFS prediction of patients with ACC.

## Data Availability

The datasets presented in this study can be found in online repositories. The names of the repository/repositories and accession number(s) can be found in the article/Supplementary Material.
