# Supplementary material for: Identification of Four Novel Prognostic Biomarkers and Construction of Two Nomograms in Adrenocortical Carcinoma: A Multi-Omics Data Study via Bioinformatics and Machine Learning Methods
Source: Front Mol Biosci. 2022 May 25;9:878073. doi: 10.3389/fmolb.2022.878073 (PMC9174903; doi:10.3389/fmolb.2022.878073)
Supplement: Supplementary file 1 [file DataSheet1.docx]

Supplementary materials:

Supplementary figures:

Figure S1: Flow diagram of data preparation, processing, analysis, and validation in this study.


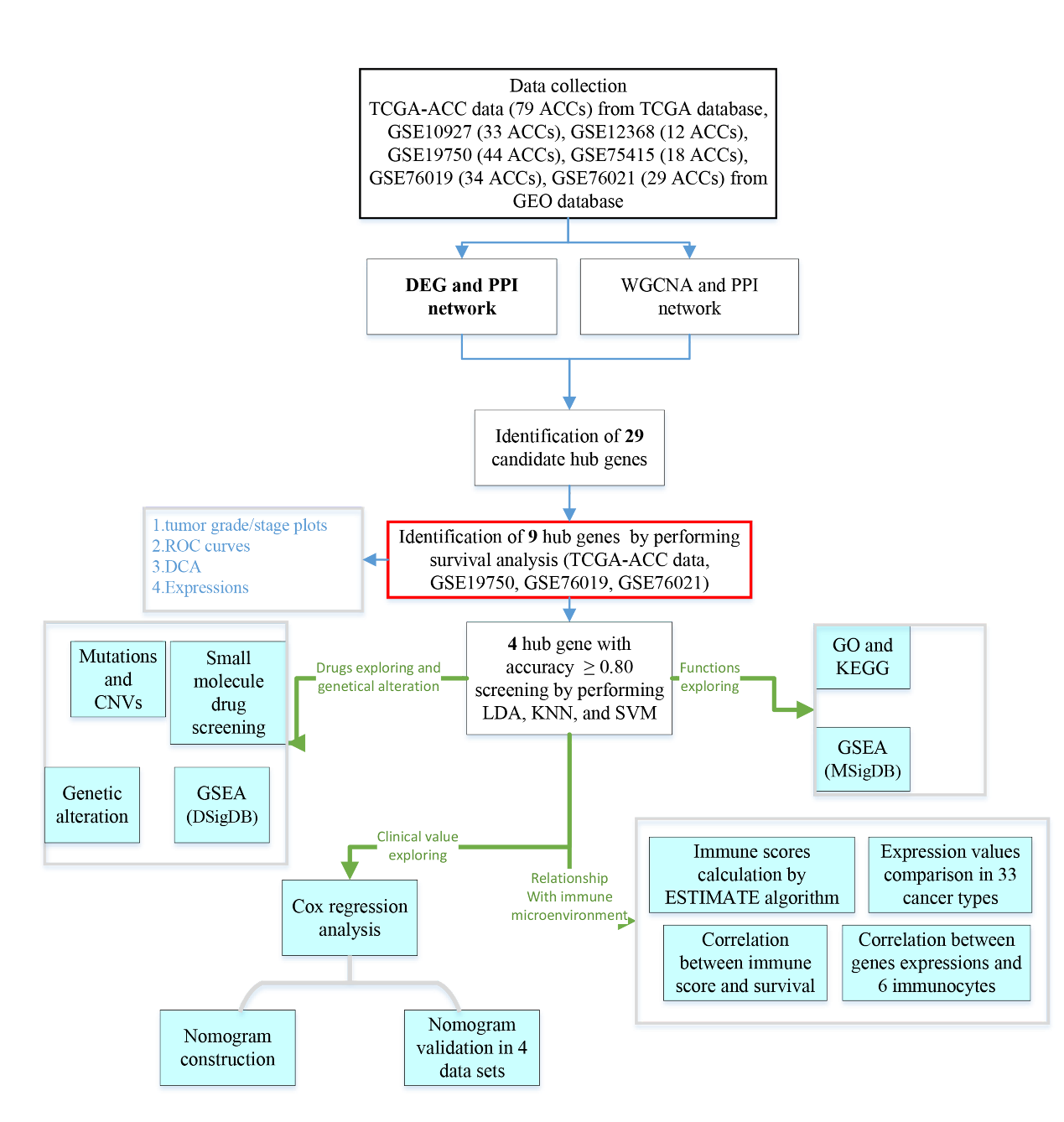


Figure S2: (A) Samples clustering to detect outliers (GSE76021). (B) The clustering was based on the expression data of GSE76021. All genes were used for the analysis by WGCNA. The color intensity was proportional to tumor stage, survival years (survival time), and survival status. Determination of soft-thresholding power in the weighted gene co-expression network analysis (WGCNA). (C) Analysis of the scale-free ﬁt index for various soft-thresholding powers (β). (D) Analysis of the mean connectivity for various soft-thresholding powers. (R) Histogram of connectivity distribution when β = 9. (F) Checking the scale free topology when β = 9. (G) The cluster dendrogram of genes in GSE76021. Each branch in the figure represents one gene, and every color below represents one co-expression module.


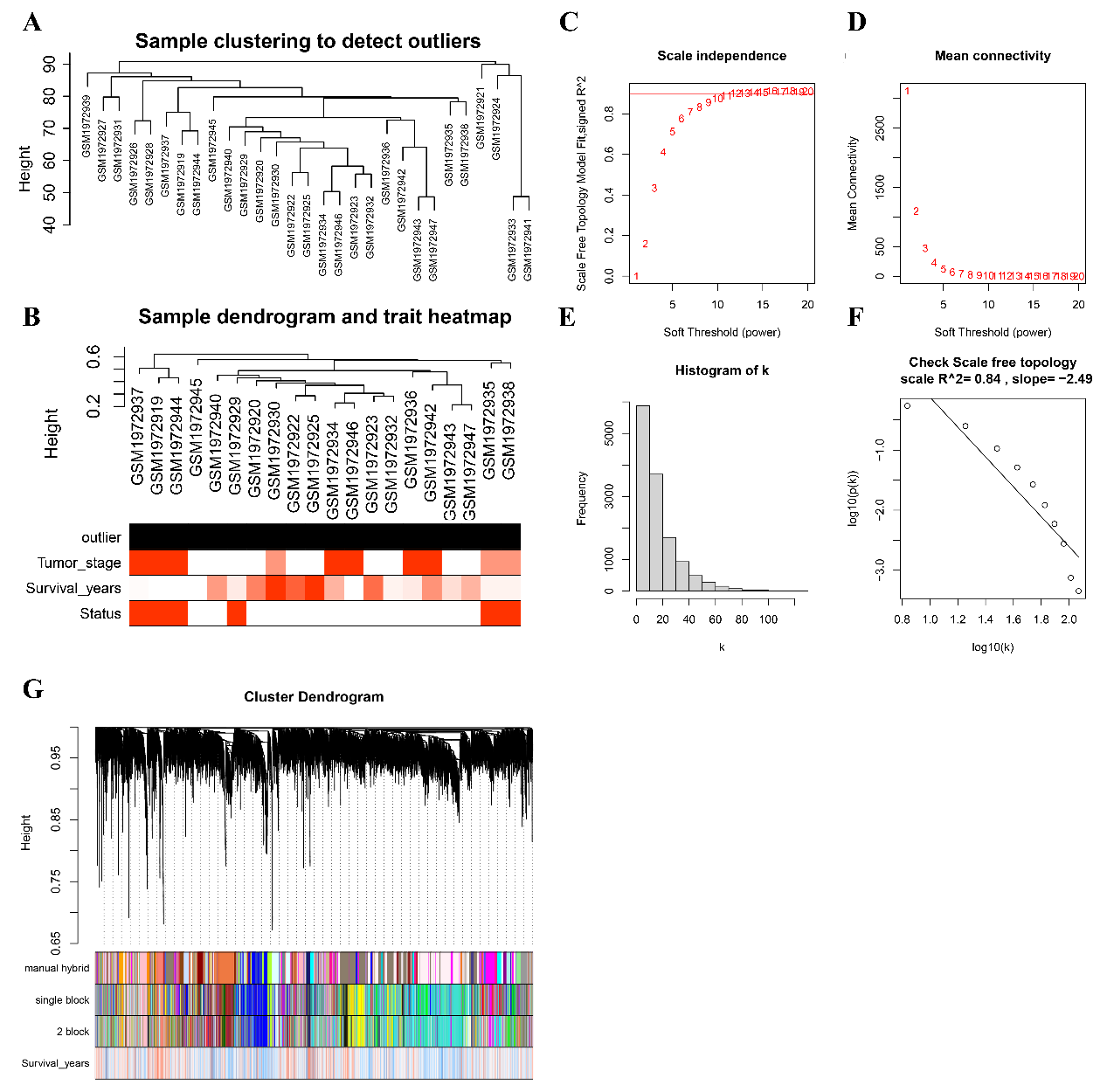


Figure S3: (A) Protein-protein interaction network of all the DEGs. (B) Protein-protein interaction network of all the genes in the blue module. (C) Overlapped hub genes in key modules and DEGs.


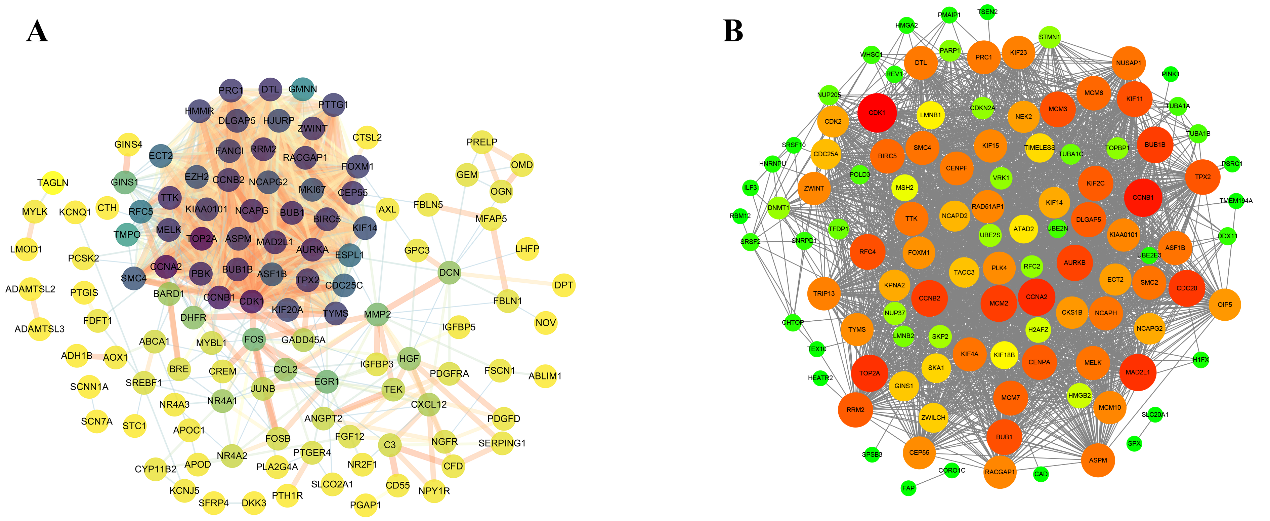


Figure S4: Disease-free survival analyses on hub genes (ASPM (A), BIRC5 (B), CCNB2 (C), CDK1 (D), DLGAP5 (E), FOXM1 (F), RACGAP1 (G), TOP2A (H), TPX2 (I)) based on TCGA-ACC data. Survival curves for patients in different groups. Red lines represent high expression of hub genes, while blue lines represent low expression of hub genes.


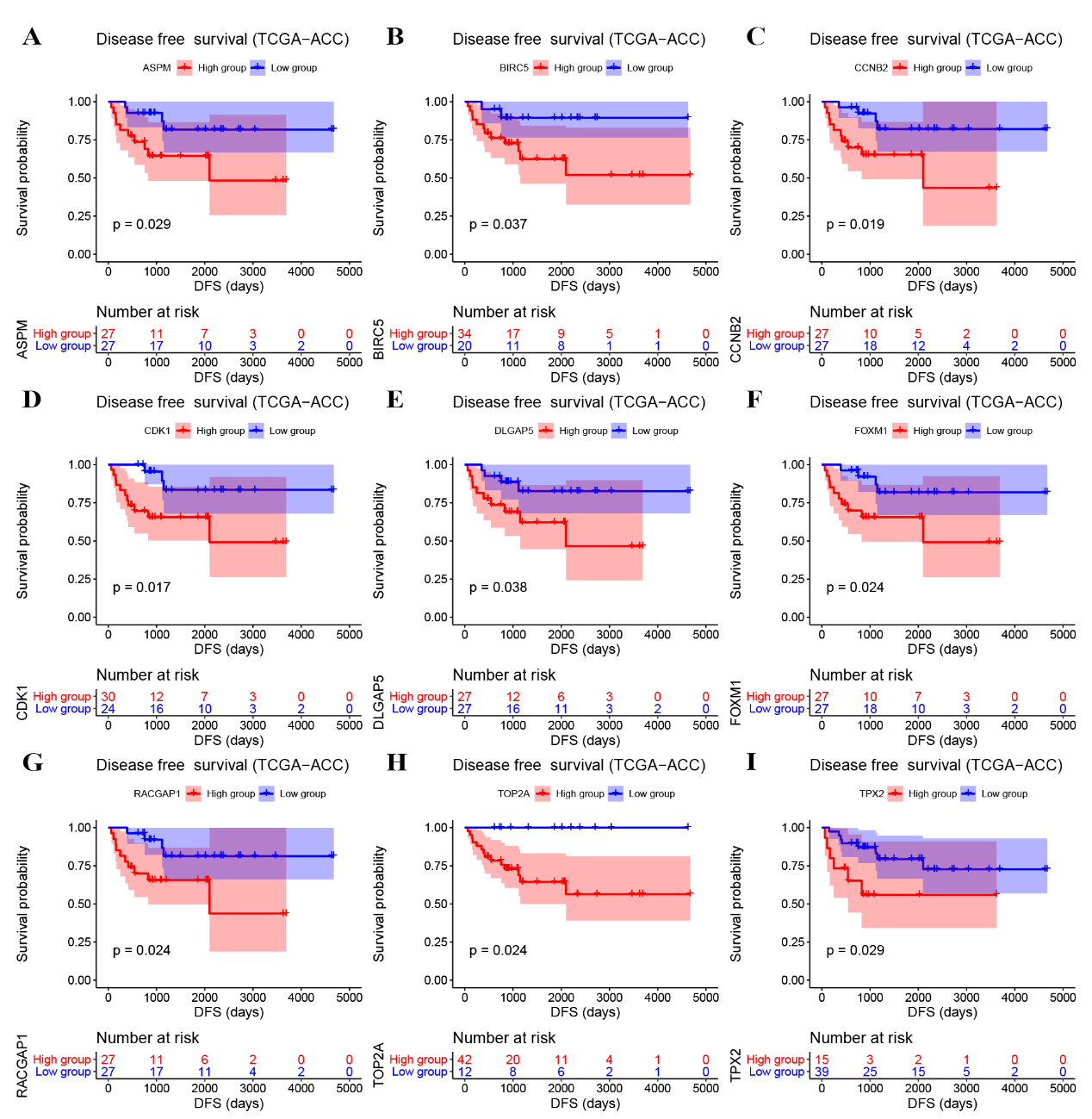


Figure S5: Overall survival analyses on hub genes (ASPM (A), BIRC5 (B), CCNB2 (C), CDK1 (D), DLGAP5 (E), FOXM1 (F), RACGAP1 (G), TOP2A (H), TPX2 (I)) based on GSE19750. Survival curves for patients in different groups. Red lines represent high expression of hub genes, while blue lines represent low expression of hub genes.


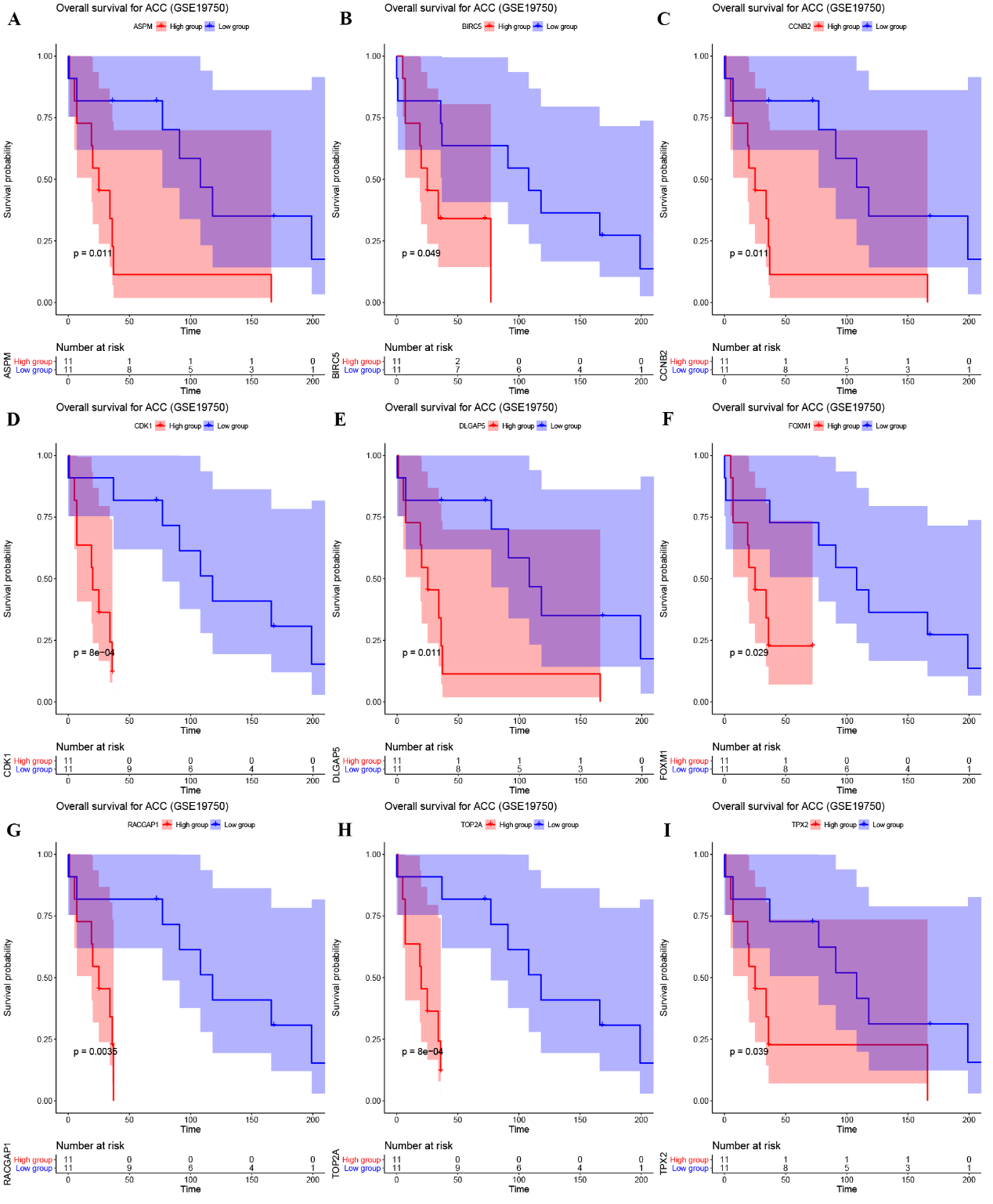


Figure S6: Event-free survival analyses on hub genes (ASPM (A), BIRC5 (B), CCNB2 (C), CDK1 (D), DLGAP5 (E), FOXM1 (F), RACGAP1 (G), TOP2A (H), TPX2 (I)) based on GSE76019. Survival curves for patients in different groups. Red lines represent high expression of hub genes, while blue lines represent low expression of hub genes.


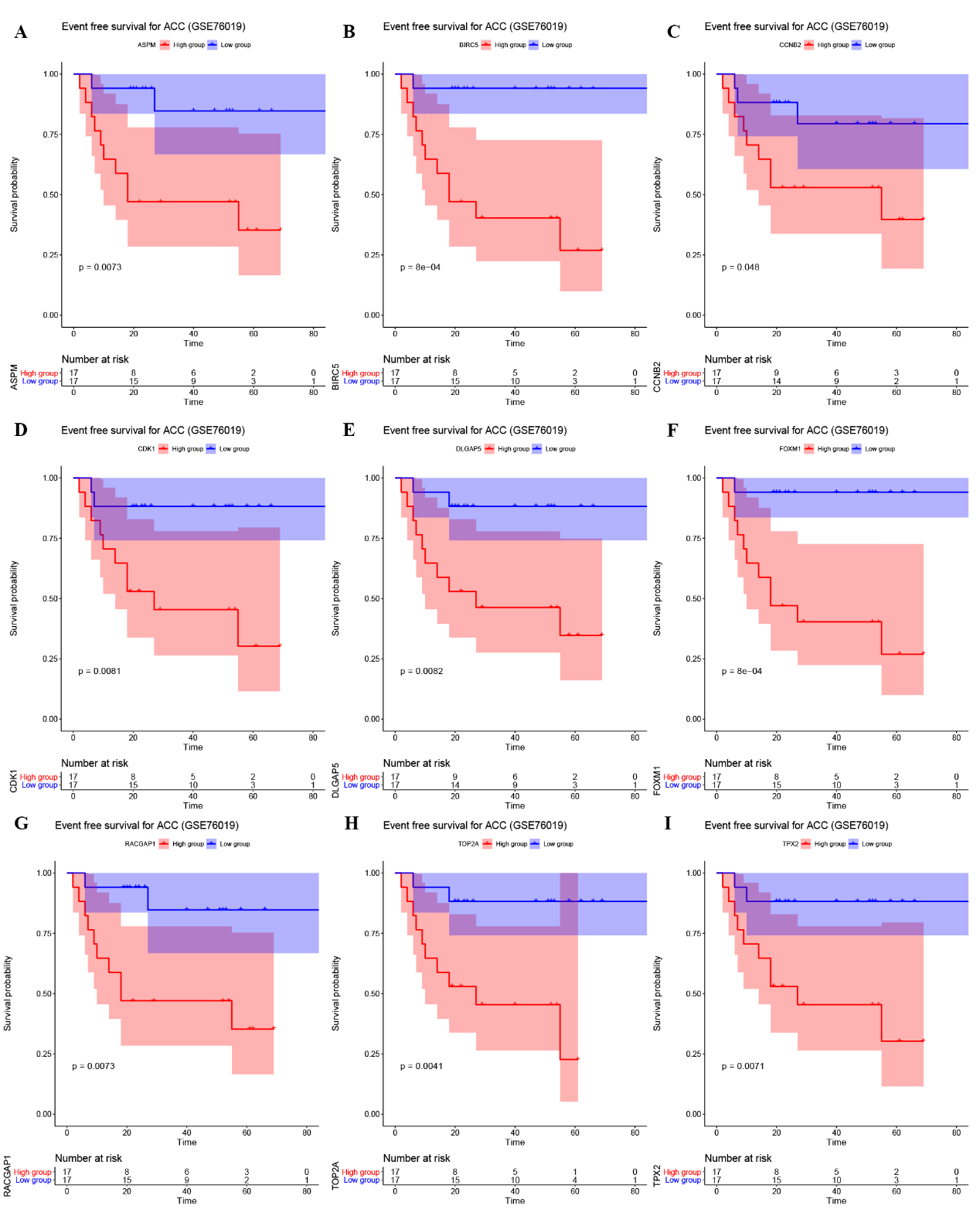


Figure S7: Event-free survival analyses on hub genes (ASPM (A), BIRC5 (B), CCNB2 (C), CDK1 (D), DLGAP5 (E), FOXM1 (F), RACGAP1 (G), TOP2A (H), TPX2 (I)) based on GSE76021. Survival curves for patients in different groups. Red lines represent high expression of hub genes, while blue lines represent low expression of hub genes.


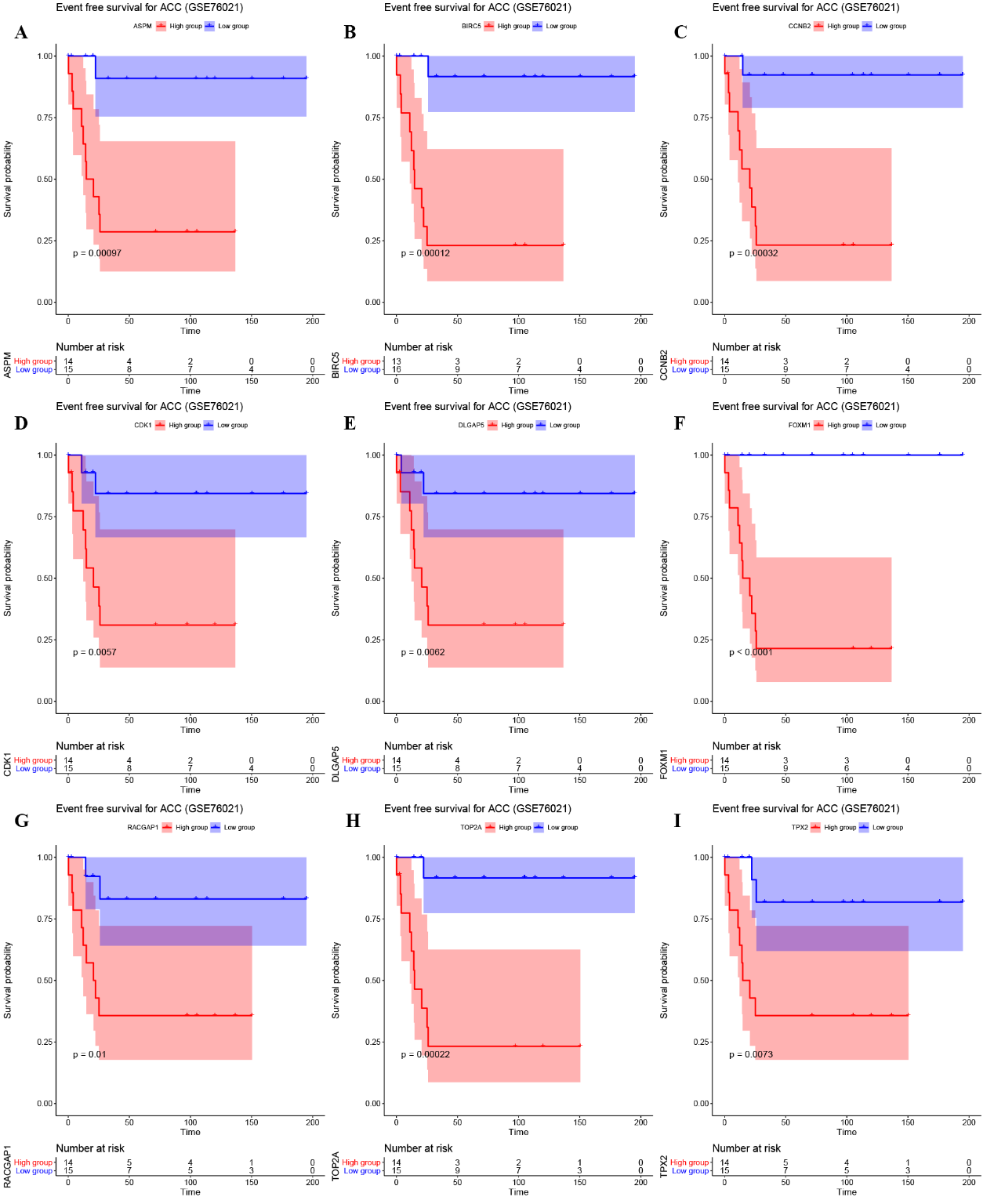


Figure S8: Decision curve analysis (DCA) for assessment of the clinical utility of hub genes (ASPM (A), BIRC5 (B), CCNB2 (C), CDK1 (D), DLGAP5 (E), FOXM1 (F), RACGAP1 (G), TOP2A (H), TPX2 (I)). The x‐axis represents the percentage of threshold probability, and the y‐axis represents the net benefit.


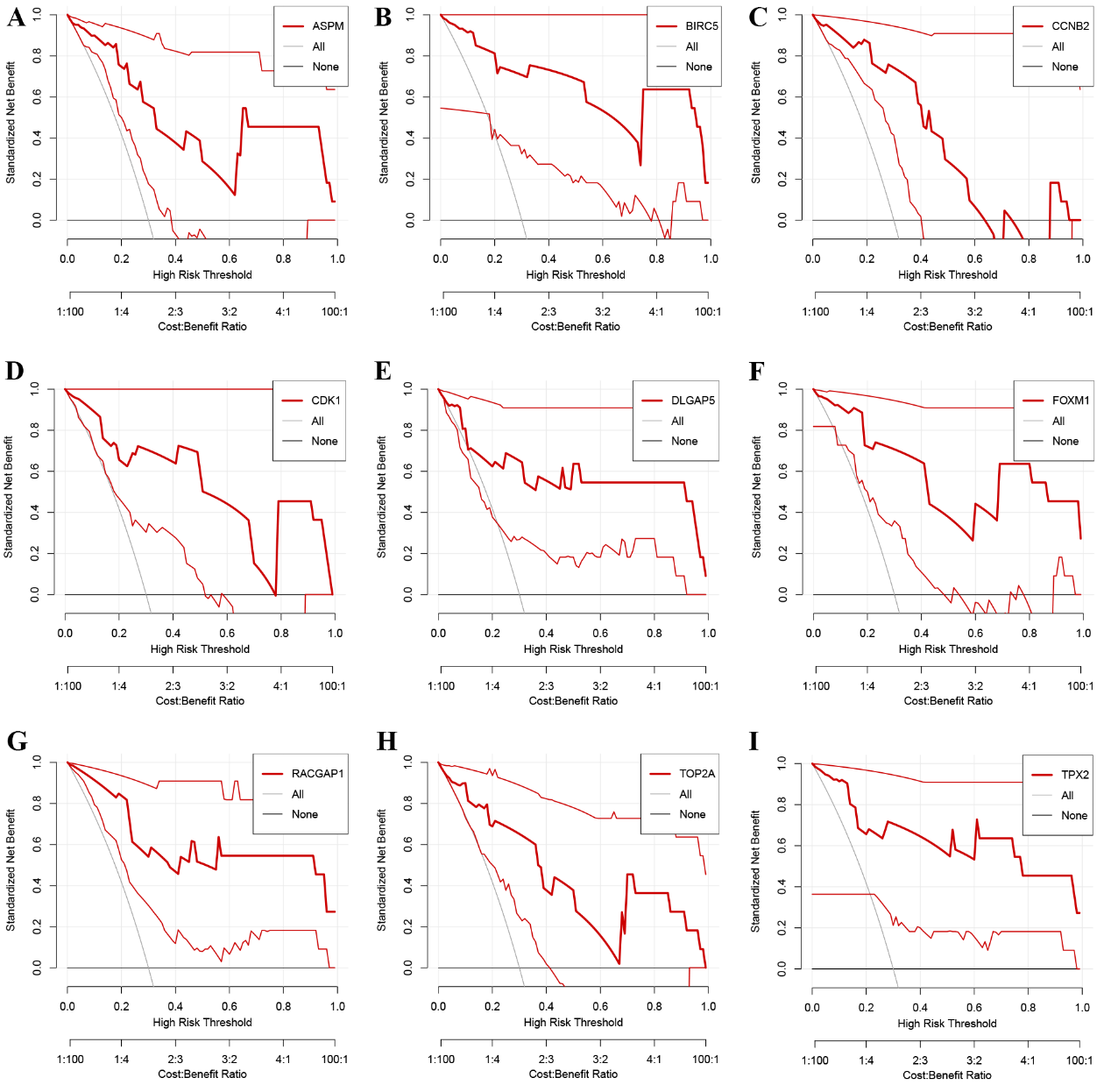


Figure S9: Bioinformatics analysis of candidate hub genes. (A) Biological process of candidate hub genes. (B) KEGG pathway enrichment of candidate hub genes.


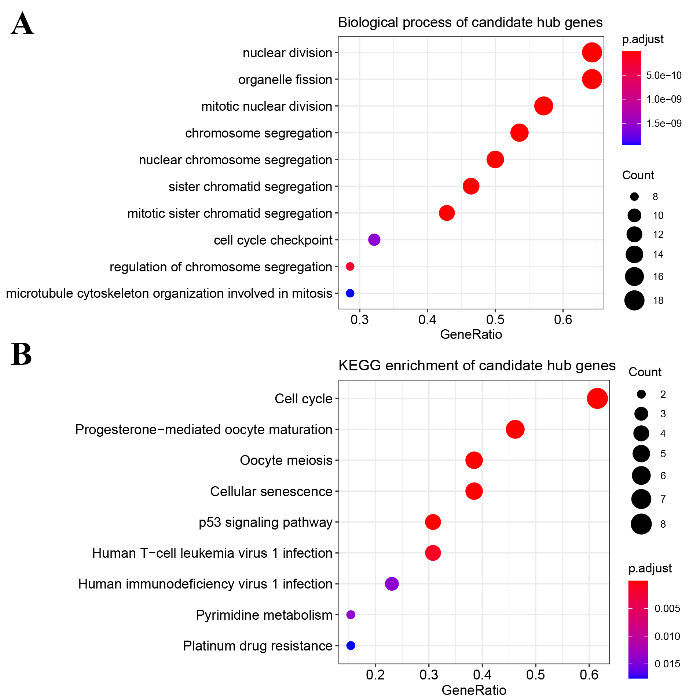


Figure S10: Correlation of MPBs expression with immune inﬁltration level in ACC. (A) ASPM expression is signiﬁcantly positively related to tumor purity and inﬁltrating levels of B cells, and dendritic cells. (B) BIRC5 expression has no signiﬁcant correlations with tumor purity and inﬁltrating levels of B cells, CD8+ T cells, CD4+ T cells, macrophages, neutrophils, and dendritic cells. (C) CCNB2 expression is signiﬁcantly positively related to tumor purity and had signiﬁcant positive correlations with inﬁltrating levels of dendritic cells. (D) CDK1 expression is signiﬁcantly positively related to tumor purity.


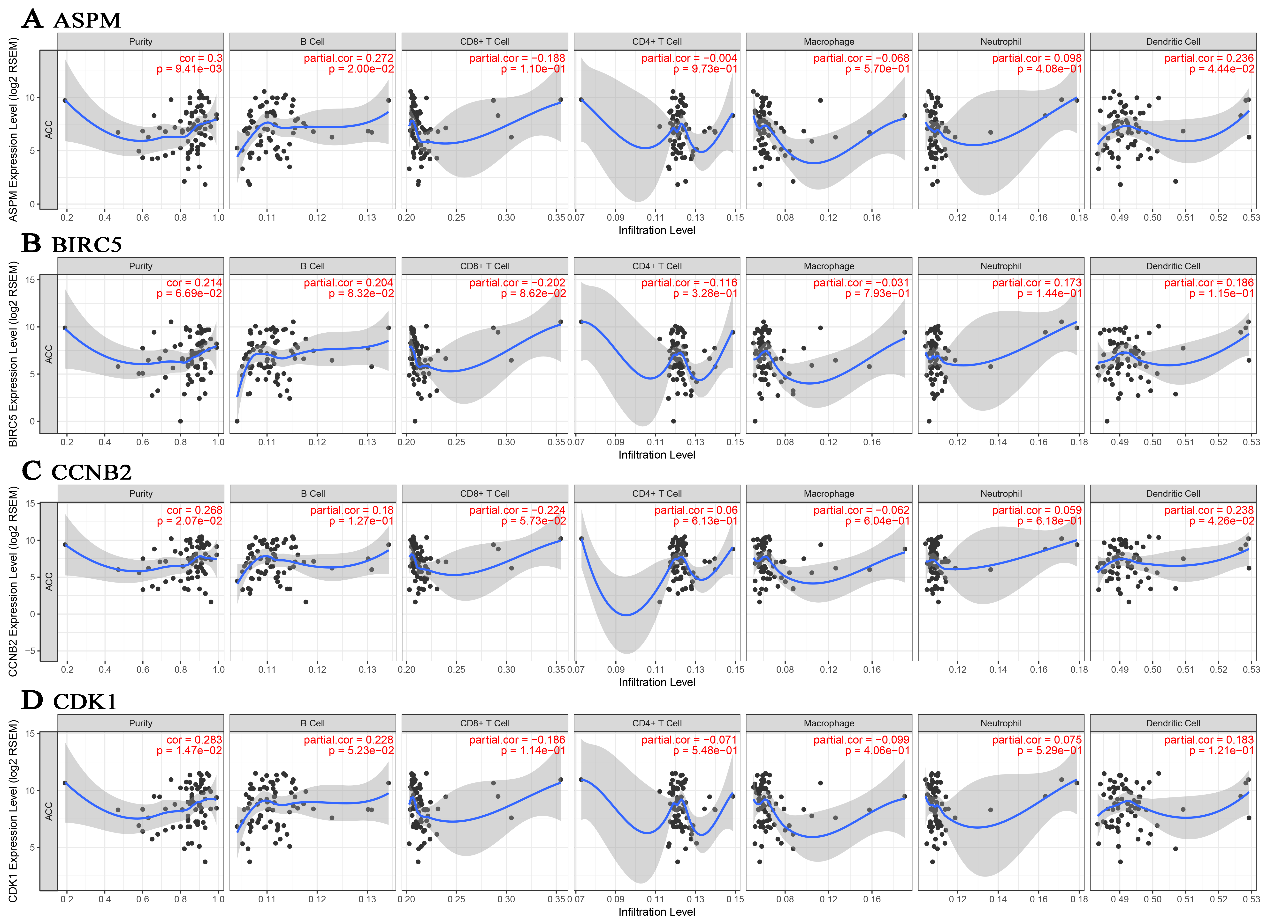


Figure S11: Human MPBs (ASPM (A), BIRC5 (B), CCNB2 (C), CDK1 (D)) expression levels in different tumor types from TCGA database were determined by TIMER (*P < 0.05, **P < 0.01, ***P < 0.001).


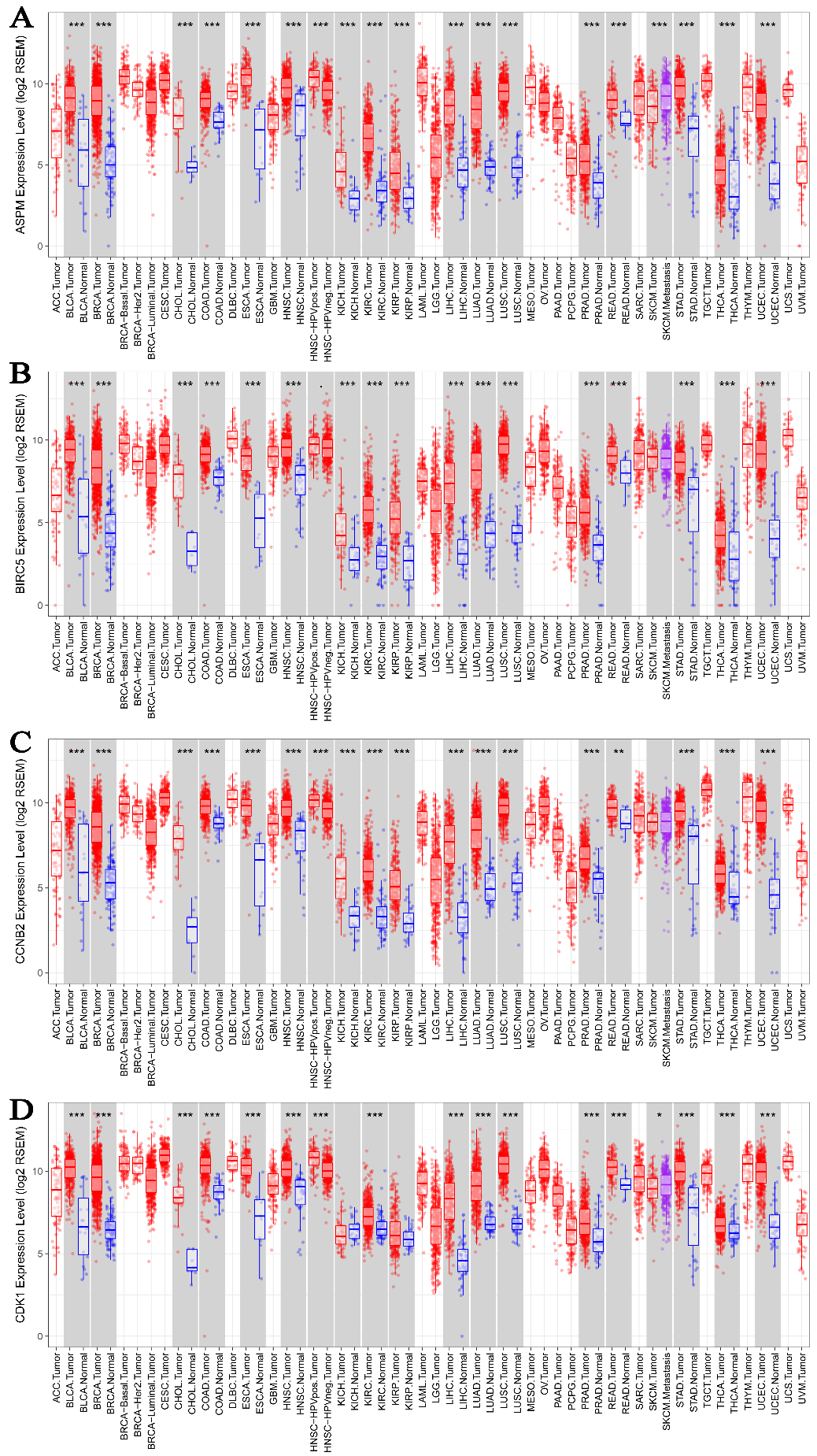


Figure S12: Schoenfeld individual test for investigating the proportional hazards assumption in OS (A) and DFS (B) Cox model.


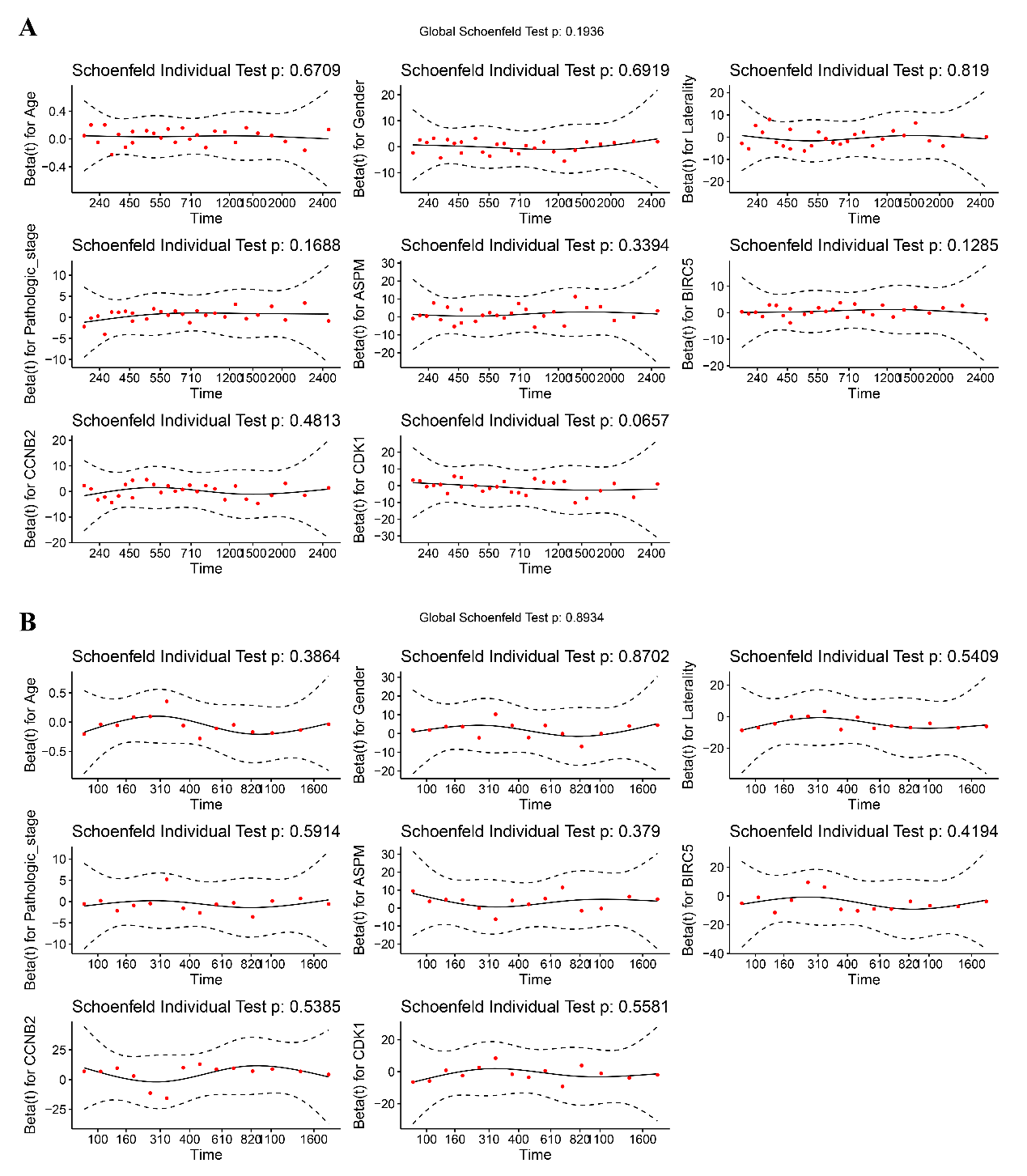


Supplementary tables:

Table S1: Gene expression microarray datasets related to ACC.

|  | Accession number of the dataset | Organization name | Platform | Status | Organism | Experiment type | Disease type | | |
| --- | --- | --- | --- | --- | --- | --- | --- | --- | --- |
|  |  |  |  |  |  |  | ACC | ACA | control |
|  | GSE10927 | University of Michigan | GPL570 | Public on Jan. 22, 2009 | Homo sapiens | Expression profiling by array | 33 | 22 | 10 |
|  | GSE12368 | Kolling Institute of Medical Research | GPL570 | Public on Jul. 29, 2009 | Homo sapiens | Expression profiling by array | 12 | 16 | 6 |
| mRNA | GSE19750 | Arizona State University | GPL570 | Public on Nov. 21, 2013 | Homo sapiens | Expression profiling by array | 44 |  | 4 |
|  | GSE75415 | St. Jude Children's Research Hospital | GPL96 | Public on Nov. 25, 2015 | Homo sapiens | Expression profiling by array | 18 | 5 | 7 |
|  | GSE76019 | St. Jude Children's Research Hospital | GPL13158 | Public on Dec. 14, 2016 | Homo sapiens | Expression profiling by array | 34 |  |  |
|  | GSE76021 | St. Jude Children's Research Hospital | GPL96 | Public on Dec. 14, 2016 | Homo sapiens | Expression profiling by array | 29 |  |  |
|  | TCGA-ACC |  |  |  |  |  | 79 |  |  |

Note: ACC: adrenocortical carcinoma; ACA: adrenocortical adenomas.

**Table S2：The overlapped DEGs in GSE75415 and GSE12368.**

|  | **GSE75415** | | **GSE12368** | |  |
| --- | --- | --- | --- | --- | --- |
| **Gene.symbol** | **adj.P.Val** | **logFC** | **adj.P.Val** | **logFC** | **Gene.title** |
| ABCA1 | 0.029684 | -2.59174 | 0.031116 | -2.05455 | ATP binding cassette subfamily A member 1 |
| ABLIM1 | 0.003284 | -2.12095 | 0.005086 | -1.71064 | actin binding LIM protein 1 |
| ABTB2 | 0.026723 | -1.54921 | 0.022875 | -1.67805 | ankyrin repeat and BTB domain containing 2 |
| ADAMTSL2 | 0.031888 | -1.73177 | 0.000439 | -4.51697 | ADAMTS like 2 |
| ADAMTSL3 | 0.004057 | -1.76792 | 0.000555 | -2.4133 | ADAMTS like 3 |
| ADH1B | 0.048797 | -1.76401 | 0.002389 | -4.60043 | alcohol dehydrogenase 1B (class I), beta polypeptide |
| AEBP1 | 0.013092 | -2.31989 | 0.023898 | -2.17828 | AE binding protein 1 |
| ANGPT2 | 0.000137 | 3.276611 | 0.000454 | 3.767116 | angiopoietin 2 |
| AOX1 | 0.001925 | -2.91202 | 0.017889 | -2.7801 | aldehyde oxidase 1 |
| APOBEC3B | 0.030004 | 2.327342 | 0.04533 | 2.664955 | apolipoprotein B mRNA editing enzyme catalytic subunit 3B |
| APOC1 | 0.001406 | -2.10176 | 0.01628 | -2.52048 | apolipoprotein C1 |
| APOD | 0.000197 | -2.20649 | 0.005428 | -2.68709 | apolipoprotein D |
| ASF1B | 0.016783 | 1.609886 | 0.014782 | 2.770606 | anti-silencing function 1B histone chaperone |
| ASPM | 0.00355 | 4.100132 | 0.007482 | 4.807382 | abnormal spindle microtubule assembly |
| ATP1B3 | 0.0087 | -1.54681 | 0.014123 | -1.92559 | ATPase Na+/K+ transporting subunit beta 3 |
| AURKA | 0.000803 | 2.708116 | 0.021598 | 2.440948 | aurora kinase A |
| AXL | 0.001704 | -2.0435 | 0.042298 | -1.7824 | AXL receptor tyrosine kinase |
| BARD1 | 3.6E-05 | 3.658095 | 0.010771 | 2.310984 | BRCA1 associated RING domain 1 |
| BIRC5 | 0.013274 | 2.377955 | 0.046872 | 2.64624 | baculoviral IAP repeat containing 5 |
| BRE | 0.041791 | -1.60948 | 0.002707 | -1.92321 | brain and reproductive organ-expressed (TNFRSF1A modulator) |
| BUB1 | 0.001929 | 3.019976 | 0.026935 | 3.966643 | BUB1 mitotic checkpoint serine/threonine kinase |
| BUB1B | 0.005943 | 1.905001 | 0.02481 | 2.345215 | BUB1 mitotic checkpoint serine/threonine kinase B |
| C3 | 0.000835 | -4.27605 | 0.026867 | -3.38875 | complement component 3 |
| C7 | 0.037064 | -3.45397 | 0.006452 | -3.92909 | complement component 7 |
| CCL2 | 0.002876 | -2.94765 | 0.013382 | -3.0382 | C-C motif chemokine ligand 2 |
| CCNA2 | 0.018874 | 1.503715 | 0.008494 | 3.001859 | cyclin A2 |
| CCNB1 | 0.014044 | 1.792258 | 0.004068 | 2.750525 | cyclin B1 |
| CCNB2 | 0.004651 | 2.143343 | 0.008209 | 3.831058 | cyclin B2 |
| CD55 | 0.020368 | -1.71863 | 0.029952 | -1.50323 | CD55 molecule (Cromer blood group) |
| CDC25C | 0.010956 | 2.475295 | 0.010756 | 3.608581 | cell division cycle 25C |
| CDK1 | 8.38E-05 | 4.13039 | 0.021596 | 3.724801 | cyclin dependent kinase 1 |
| CEP55 | 0.033642 | 2.520884 | 0.022619 | 3.504322 | centrosomal protein 55 |
| CFD | 0.003401 | -3.21568 | 0.025706 | -2.12143 | complement factor D |
| CHRDL1 | 0.008775 | -2.61694 | 0.03237 | -3.59785 | chordin like 1 |
| COQ8A | 0.003836 | -1.96094 | 0.002991 | -1.7006 | coenzyme Q8A |
| CORO2B | 0.017261 | -1.74054 | 0.025324 | -2.33623 | coronin 2B |
| CREBL2 | 0.002463 | -1.76387 | 0.025086 | -1.50672 | cAMP responsive element binding protein like 2 |
| CREM | 0.000774 | -1.71456 | 0.006728 | -1.71874 | cAMP responsive element modulator |
| CRHBP | 0.001713 | -1.8812 | 0.007371 | -4.27346 | corticotropin releasing hormone binding protein |
| CRISPLD2 | 0.011998 | -1.65282 | 0.007964 | -1.89002 | cysteine rich secretory protein LCCL domain containing 2 |
| CTH | 0.045749 | -1.60164 | 0.009245 | -2.05555 | cystathionine gamma-lyase |
| CTSV | 0.030702 | 2.234132 | 0.025508 | 1.743382 | cathepsin V |
| CXCL12 | 0.000315 | -3.00351 | 0.010363 | -3.77837 | C-X-C motif chemokine ligand 12 |
| CYP11B2 | 8.38E-05 | -4.94874 | 0.000909 | -4.98744 | cytochrome P450 family 11 subfamily B member 2 |
| DCN | 0.000592 | -3.37234 | 0.00383 | -2.88741 | decorin |
| DEPTOR | 0.045602 | -1.50893 | 0.042707 | -2.18774 | DEP domain containing MTOR-interacting protein |
| DHFR | 0.011758 | 1.587903 | 0.021134 | 1.622167 | dihydrofolate reductase |
| DKK3 | 0.028182 | -2.61606 | 0.015551 | -2.06417 | dickkopf WNT signaling pathway inhibitor 3 |
| DLGAP5 | 0.005292 | 2.624377 | 0.028952 | 3.222416 | DLG associated protein 5 |
| DPT | 0.018235 | -1.85223 | 0.011912 | -4.04217 | dermatopontin |
| DTL | 0.00059 | 3.499813 | 0.006144 | 4.317546 | denticleless E3 ubiquitin protein ligase homolog |
| ECT2 | 0.013274 | 2.159048 | 0.003193 | 2.139256 | epithelial cell transforming 2 |
| EGR1 | 0.005891 | -2.08833 | 0.031378 | -1.52846 | early growth response 1 |
| EMCN | 0.031445 | -2.12633 | 0.033719 | -1.53688 | endomucin |
| ENC1 | 0.024495 | 2.26288 | 0.002567 | 2.764433 | ectodermal-neural cortex 1 |
| EPHX2 | 0.008176 | -2.74896 | 0.00838 | -2.03518 | epoxide hydrolase 2 |
| ESPL1 | 0.035273 | 1.830645 | 0.049953 | 1.713663 | extra spindle pole bodies like 1, separase |
| EZH2 | 0.00496 | 2.351576 | 0.018462 | 2.038998 | enhancer of zeste 2 polycomb repressive complex 2 subunit |
| FANCI | 0.000111 | 2.880655 | 0.007817 | 2.999237 | Fanconi anemia complementation group I |
| FBLN1 | 0.000056 | -2.63628 | 0.023348 | -3.1522 | fibulin 1 |
| FBLN5 | 0.003731 | -3.08691 | 0.007758 | -3.31184 | fibulin 5 |
| FDFT1 | 0.020038 | 1.837241 | 0.041131 | 2.439584 | farnesyl-diphosphate farnesyltransferase 1 |
| FGF12 | 0.020319 | -1.80979 | 0.034153 | -3.23274 | fibroblast growth factor 12 |
| FLVCR2 | 0.032961 | -1.77122 | 0.024076 | -1.58092 | feline leukemia virus subgroup C cellular receptor family member 2 |
| FMO2 | 5.41E-05 | -2.042 | 0.000541 | -4.2948 | flavin containing monooxygenase 2 |
| FOS | 0.000844 | -3.21912 | 0.030348 | -2.47507 | Fos proto-oncogene, AP-1 transcription factor subunit |
| FOSB | 0.004144 | -4.00814 | 0.031684 | -3.50256 | FosB proto-oncogene, AP-1 transcription factor subunit |
| FOXM1 | 0.007704 | 3.160484 | 0.014308 | 3.956482 | forkhead box M1 |
| FSCN1 | 0.017366 | 2.362547 | 0.016978 | 1.733118 | fascin actin-bundling protein 1 |
| GADD45A | 0.036526 | 1.55324 | 0.014436 | 1.640124 | growth arrest and DNA damage inducible alpha |
| GEM | 0.00116 | -2.12071 | 0.021266 | -2.29492 | GTP binding protein overexpressed in skeletal muscle |
| GINS1 | 0.036095 | 2.008893 | 0.01139 | 2.242871 | GINS complex subunit 1 |
| GINS4 | 0.041133 | 1.90038 | 0.013533 | 1.689863 | GINS complex subunit 4 |
| GMNN | 0.008297 | 1.644224 | 0.01261 | 2.014307 | geminin, DNA replication inhibitor |
| GPC3 | 0.03587 | -1.93452 | 0.010756 | -2.10471 | glypican 3 |
| GPM6B | 0.045438 | -1.96843 | 0.017196 | -1.94108 | glycoprotein M6B |
| HGF | 0.002442 | -2.65876 | 0.012612 | -3.65289 | hepatocyte growth factor |
| HJURP | 0.018437 | 2.394495 | 0.023705 | 3.284476 | Holliday junction recognition protein |
| HMMR | 0.004581 | 2.769855 | 0.033374 | 3.62667 | hyaluronan mediated motility receptor |
| HOMER1 | 0.003284 | 3.1481 | 0.002261 | 2.447876 | homer scaffolding protein 1 |
| HOPX | 0.000699 | -3.51825 | 0.010756 | -3.28212 | HOP homeobox |
| HOXA5 | 0.003305 | -2.12358 | 0.000233 | -3.22094 | homeobox A5 |
| IGFBP3 | 0.044714 | 1.925274 | 0.011912 | 2.525573 | insulin like growth factor binding protein 3 |
| IGFBP5 | 0.002809 | -1.63049 | 0.007052 | -1.70336 | insulin like growth factor binding protein 5 |
| IGFBP6 | 0.000111 | -2.85807 | 0.000555 | -3.0896 | insulin like growth factor binding protein 6 |
| ITGA8 | 4.87E-05 | -2.50516 | 0.021149 | -1.61484 | integrin subunit alpha 8 |
| JUNB | 4.08E-05 | -2.26492 | 0.000698 | -2.49416 | JunB proto-oncogene, AP-1 transcription factor subunit |
| KCNJ5 | 0.008333 | -2.37601 | 0.032322 | -2.61354 | potassium voltage-gated channel subfamily J member 5 |
| KCNQ1 | 3.6E-05 | -3.64645 | 0.018409 | -3.17376 | potassium voltage-gated channel subfamily Q member 1 |
| KIAA0101 | 0.00346 | 2.848629 | 0.017103 | 3.509931 | KIAA0101 |
| KIAA1024 | 0.028304 | -2.29267 | 0.010771 | -5.27861 | KIAA1024 |
| KIF14 | 0.003836 | 1.641577 | 0.011912 | 3.779868 | kinesin family member 14 |
| KIF20A | 0.000712 | 3.781286 | 0.012947 | 4.315924 | kinesin family member 20A |
| LHFP | 0.003698 | -1.62323 | 0.019409 | -1.66113 | lipoma HMGIC fusion partner |
| LMOD1 | 0.002968 | -3.59813 | 0.008184 | -2.45584 | leiomodin 1 |
| LOC100130872///SPON2 | 0.008839 | -1.77444 | 0.008547 | -1.70848 | uncharacterized LOC100130872///spondin 2 |
| LUZP2 | 0.014289 | -2.39087 | 0.046872 | -2.0522 | leucine zipper protein 2 |
| MAD2L1 | 0.046844 | 1.639392 | 0.001941 | 2.728025 | MAD2 mitotic arrest deficient-like 1 (yeast) |
| MELK | 0.002794 | 2.627104 | 0.016319 | 3.518353 | maternal embryonic leucine zipper kinase |
| MFAP5 | 0.02766 | -1.83214 | 0.007482 | -3.03855 | microfibrillar associated protein 5 |
| MKI67 | 0.011758 | 2.293719 | 0.040908 | 1.87957 | marker of proliferation Ki-67 |
| MMP2 | 8.62E-05 | -2.5873 | 0.007482 | -2.84216 | matrix metallopeptidase 2 |
| MYBL1 | 0.044714 | 2.002958 | 0.005934 | 2.61827 | MYB proto-oncogene like 1 |
| MYLK | 0.003401 | -1.96604 | 0.035205 | -1.9186 | myosin light chain kinase |
| NAV3 | 0.044969 | -2.52805 | 0.008184 | -2.6298 | neuron navigator 3 |
| NCAPG | 0.03747 | 2.691438 | 0.031461 | 2.506863 | non-SMC condensin I complex subunit G |
| NCAPG2 | 0.007844 | 1.879704 | 0.022668 | 1.925002 | non-SMC condensin II complex subunit G2 |
| NGFR | 0.01278 | -2.03593 | 0.038863 | -2.93028 | nerve growth factor receptor |
| NOV | 0.000687 | -3.95959 | 0.047479 | -4.52782 | nephroblastoma overexpressed |
| NPY1R | 0.015872 | -3.12518 | 0.003846 | -4.97194 | neuropeptide Y receptor Y1 |
| NR2F1 | 0.000104 | -3.27373 | 0.002229 | -2.91958 | nuclear receptor subfamily 2 group F member 1 |
| NR2F2///NR2F1 | 0.002952 | -2.35076 | 0.010021 | -1.72959 | nuclear receptor subfamily 2 group F member 2///nuclear receptor subfamily 2 group F member 1 |
| NR4A1 | 0.00064 | -2.95971 | 0.032722 | -1.68106 | nuclear receptor subfamily 4 group A member 1 |
| NR4A2 | 9.63E-05 | -4.06258 | 0.00253 | -4.733 | nuclear receptor subfamily 4 group A member 2 |
| NR4A3 | 0.011586 | -1.69812 | 0.046824 | -2.44231 | nuclear receptor subfamily 4 group A member 3 |
| OGN | 0.019783 | -2.62846 | 0.007067 | -4.19677 | osteoglycin |
| OLFML3 | 0.003401 | -3.29892 | 0.010161 | -2.56718 | olfactomedin like 3 |
| OMD | 0.0062 | -2.50784 | 0.014809 | -3.60279 | osteomodulin |
| PBK | 0.011998 | 2.167052 | 0.013614 | 3.561479 | PDZ binding kinase |
| PCSK2 | 0.015872 | -2.08445 | 0.011305 | -4.73656 | proprotein convertase subtilisin/kexin type 2 |
| PDGFD | 0.000844 | -2.67278 | 0.020341 | -3.5622 | platelet derived growth factor D |
| PDGFRA | 3.6E-05 | -3.84046 | 0.013614 | -4.8096 | platelet derived growth factor receptor alpha |
| PGAP1 | 0.044116 | -2.42052 | 0.004954 | -1.93757 | post-GPI attachment to proteins 1 |
| PLA2G4A | 0.00821 | -2.65881 | 0.003204 | -3.25562 | phospholipase A2 group IVA |
| PLP1 | 0.024651 | -1.93562 | 0.011305 | -3.1931 | proteolipid protein 1 |
| PRC1 | 0.000328 | 3.834841 | 0.018885 | 3.037351 | protein regulator of cytokinesis 1 |
| PRELP | 0.000315 | -3.21151 | 0.006107 | -2.08174 | proline and arginine rich end leucine rich repeat protein |
| PTGER4 | 0.016789 | -2.64439 | 0.004828 | -3.33135 | prostaglandin E receptor 4 |
| PTGIS | 3.6E-05 | -2.8269 | 0.029109 | -2.24047 | prostaglandin I2 (prostacyclin) synthase |
| PTH1R | 1.28E-05 | -2.06778 | 0.000542 | -2.36262 | parathyroid hormone 1 receptor |
| PTTG1 | 0.005275 | 1.884421 | 0.033096 | 2.738403 | pituitary tumor-transforming 1 |
| PTTG3P | 0.006106 | 2.103834 | 0.007482 | 3.112747 | pituitary tumor-transforming 3, pseudogene |
| RACGAP1 | 0.004513 | 1.911264 | 0.022378 | 2.633375 | Rac GTPase activating protein 1 |
| RAI2 | 0.003285 | -3.28202 | 0.049817 | -2.52358 | retinoic acid induced 2 |
| RAPGEF4 | 0.024295 | -2.01725 | 0.017913 | -2.68203 | Rap guanine nucleotide exchange factor 4 |
| RFC5 | 0.015324 | 2.090284 | 0.020183 | 1.605714 | replication factor C subunit 5 |
| RRM2 | 0.004581 | 3.375718 | 0.037861 | 3.091205 | ribonucleotide reductase regulatory subunit M2 |
| SCN7A | 0.042847 | -1.98729 | 0.000319 | -5.03951 | sodium voltage-gated channel alpha subunit 7 |
| SCNN1A | 0.009933 | -1.88343 | 0.004954 | -5.81477 | sodium channel epithelial 1 alpha subunit |
| SEPT4 | 0.040674 | -1.58267 | 0.023185 | -2.77844 | septin 4 |
| SERPING1 | 0.002343 | -1.78048 | 0.016281 | -2.23027 | serpin family G member 1 |
| SFRP4 | 0.000315 | -2.00043 | 0.023185 | -2.29289 | secreted frizzled related protein 4 |
| SGCG | 0.000315 | -2.90496 | 0.010424 | -2.38947 | sarcoglycan gamma |
| SLCO2A1 | 0.000699 | -1.79761 | 0.013382 | -1.94851 | solute carrier organic anion transporter family member 2A1 |
| SMC4 | 0.034242 | 1.546071 | 0.008547 | 1.956503 | structural maintenance of chromosomes 4 |
| SORBS2 | 0.016163 | -2.79615 | 0.041131 | -3.65997 | sorbin and SH3 domain containing 2 |
| SPON1 | 0.002463 | -3.60048 | 0.002991 | -3.47053 | spondin 1 |
| SREBF1 | 0.005218 | -1.80399 | 0.022756 | -1.99028 | sterol regulatory element binding transcription factor 1 |
| STC1 | 2.71E-05 | 4.470948 | 0.001726 | 3.754324 | stanniocalcin 1 |
| TAGLN | 0.012469 | -1.64757 | 0.03853 | -2.00013 | transgelin |
| TCF21 | 0.005943 | -2.62582 | 0.008184 | -2.6026 | transcription factor 21 |
| TEK | 0.008088 | -2.30751 | 0.010039 | -2.06176 | TEK receptor tyrosine kinase |
| TENM4 | 0.010829 | -1.86467 | 0.017924 | -2.8062 | teneurin transmembrane protein 4 |
| TESC | 0.048098 | -1.65521 | 0.033199 | -1.79129 | tescalcin |
| TKFC | 0.026013 | -2.32015 | 0.003204 | -1.89203 | triokinase and FMN cyclase |
| TLE2 | 0.002323 | -1.59659 | 0.017924 | -1.74716 | transducin like enhancer of split 2 |
| TMPO | 0.022555 | 1.509363 | 0.011236 | 1.820768 | thymopoietin |
| TOP2A | 0.002022 | 3.52363 | 0.012612 | 4.364297 | topoisomerase (DNA) II alpha |
| TPX2 | 0.000924 | 2.98077 | 0.001726 | 3.640644 | TPX2, microtubule nucleation factor |
| TTK | 0.028297 | 2.410458 | 0.006364 | 3.471648 | TTK protein kinase |
| TYMS | 0.013314 | 2.50674 | 0.019773 | 2.271375 | thymidylate synthetase |
| WFDC1 | 0.005168 | -3.41875 | 0.000555 | -3.16519 | WAP four-disulfide core domain 1 |
| ZNF185 | 0.020647 | -1.99003 | 0.040902 | -1.62863 | zinc finger protein 185 (LIM domain) |
| ZNF331 | 8.38E-05 | -2.56442 | 0.000555 | -2.89673 | zinc finger protein 331 |
| ZWINT | 0.003307 | 1.82824 | 0.032994 | 2.118087 | ZW10 interacting kinetochore protein |

Table S3：Results of CMap analysis.

| **cmap name** | **mean** | **n** | **enrichment** | ***P*** | **specificity** | **percent non-null** |
| --- | --- | --- | --- | --- | --- | --- |
| trichostatin A | -0.284 | 182 | -0.193 | 0 | 0.8317 | 66 |
| thioridazine | -0.376 | 20 | -0.301 | 0.04024 | 0.4516 | 70 |
| chlorpromazine | -0.446 | 19 | -0.367 | 0.00824 | 0.0321 | 78 |
| genistein | 0.262 | 17 | 0.466 | 0.00074 | 0.0174 | 64 |
| trifluoperazine | -0.437 | 16 | -0.465 | 0.00104 | 0.1538 | 75 |
| alpha-estradiol | -0.481 | 16 | -0.377 | 0.0151 | 0.235 | 87 |
| 15-delta prostaglandin J2 | -0.46 | 15 | -0.419 | 0.00655 | 0.2181 | 66 |
| vorinostat | -0.491 | 12 | -0.435 | 0.01351 | 0.5044 | 91 |

**Table S4: Survival analysis of candidate hub genes.**

|  | TCGA-ACC OS | TCGA-ACC DFS | GSE19750 OS | GSE76019 EFS | GSE76021 EFS |
| --- | --- | --- | --- | --- | --- |
| ASF1B | 4.00E-06 | 1.80E-04 | 4.90E-02 | 6.00E-01 | 1.50E-03 |
| ASPM | < 0.0001 | 0.029 | 1.10E-02 | 7.30E-03 | 9.70E-04 |
| BIRC5 | < 0.0001 | 0.037 | 4.90E-02 | 8.00E-04 | 1.20E-04 |
| BUB1 | 2.60E-06 | 7.90E-06 | 8.30E-01 | 6.80E-04 | ＜0.0001 |
| BUB1B | < 0.0001 | 0.074 | 3.90E-02 | 1.40E-02 | 1.50E-03 |
| CCNA2 | < 0.0001 | 0.72 | 6.60E-03 | 1.40E-02 | 2.20E-03 |
| CCNB1 | 1.00E-04 | 9.50E-03 | 4.10E-01 | 4.10E-03 | 2.20E-03 |
| CCNB2 | < 0.0001 | 0.019 | 1.10E-02 | 4.80E-02 | 3.20E-04 |
| CDK1 | < 0.0001 | 0.017 | 8.00E-04 | 8.10E-03 | 5.70E-03 |
| CEP55 | 5.20E-07 | 1.30E-05 | 1.10E-04 | 6.20E-02 | 3.80E-04 |
| DLGAP5 | < 0.0001 | 0.038 | 1.10E-02 | 8.20E-03 | 6.20E-03 |
| DTL | 4.80E-05 | 3.30E-04 | 1.00E-01 | 5.00E-03 | 4.80E-04 |
| ECT2 | 1.00E-05 | 1.40E-05 | 6.20E-01 | 9.00E-02 | 3.40E-03 |
| FOXM1 | < 0.0001 | 0.024 | 2.90E-02 | 8.00E-04 | ＜0.0001 |
| GINS1 | 2.80E-04 | 3.00E-03 | 9.40E-01 | 1.60E-01 | 4.10E-03 |
| KIAA0101 | < 0.0001 | 0.1 | 1.10E-02 | 8.00E-04 | 3.20E-04 |
| KIF14 | 6.20E-06 | 1.60E-05 | 1.30E-01 | 5.00E-03 | 4.70E-03 |
| MAD2L1 | 3.80E-04 | 4.60E-03 | 1.30E-02 | 2.30E-01 | 2.00E-04 |
| MELK | 4.30E-06 | 2.30E-04 | 7.70E-03 | 7.20E-02 | 3.80E-04 |
| NCAPG2 | 1.30E-05 | 7.40E-04 | 2.60E-02 | 1.90E-01 | ＜0.0001 |
| PRC1 | < 0.0001 | 0.11 | 1.10E-02 | 5.70E-03 | ＜0.0001 |
| RACGAP1 | < 0.0001 | 0.024 | 3.50E-03 | 7.30E-03 | 1.00E-02 |
| RRM2 | < 0.0001 | 0.14 | 3.50E-03 | 4.30E-03 | 7.30E-03 |
| SMC4 | 9.70E-06 | 2.10E-05 | 8.60E-01 | 5.10E-02 | 1.30E-04 |
| TOP2A | < 0.0001 | 0.024 | 8.00E-04 | 4.10E-03 | 2.20E-04 |
| TPX2 | < 0.0001 | 0.029 | 3.90E-02 | 7.10E-03 | 7.30E-03 |
| TTK | 2.90E-04 | 8.90E-03 | 9.00E-01 | 3.50E-02 | 3.40E-03 |
| TYMS | 8.60E-04 | 9.70E-06 | 4.20E-01 | 4.60E-04 | ＜0.0001 |
| ZWINT | 1.50E-08 | 1.70E-05 | 1.00E-01 | 3.50E-02 | 1.80E-03 |

Note: OS: overall survival; DFS: disease-free survival; EFS: event-free survival.

Table S5: Cox univariable analyses of the nine genes based on several cohorts.

|  | Variable | Univariate analysis | | | |
| --- | --- | --- | --- | --- | --- |
|  |  | HR | LCI | UCI | *P* value |
| GSE76019 (EFS) | ASPM | 1.72 | 1.08 | 2.75 | 0.022 |
|  | BIRC5 | 1.57 | 1.08 | 2.27 | 0.019 |
|  | CCNB2 | 1.64 | 1.02 | 2.64 | 0.04 |
|  | CDK1 | 1.65 | 1.08 | 2.51 | 0.021 |
|  | DLGAP5 | 1.89 | 1.19 | 3.02 | 0.007 |
|  | FOXM1 | 1.61 | 1.07 | 2.41 | 0.023 |
|  | RACGAP1 | 2.08 | 1.15 | 3.75 | 0.015 |
|  | TOP2A | 1.88 | 1.17 | 3.01 | 0.009 |
|  | TPX2 | 1.78 | 1.06 | 2.97 | 0.028 |
| GSE76021  (EFS) | ASPM | 2.43 | 1.49 | 3.97 | 0 |
|  | BIRC5 | 2.29 | 1.47 | 3.57 | 0 |
|  | CCNB2 | 4.09 | 1.7 | 9.86 | 0.002 |
|  | CDK1 | 3.11 | 1.65 | 5.87 | 0 |
|  | DLGAP5 | 4.38 | 2.04 | 9.4 | 0 |
|  | FOXM1 | 5.08 | 2.23 | 11.55 | 0 |
|  | RACGAP1 | 3.4 | 1.7 | 6.81 | 0.001 |
|  | TOP2A | 1.96 | 1.32 | 2.91 | 0.001 |
|  | TPX2 | 3.32 | 1.78 | 6.17 | 0 |
| TCGA  (OS) | ASPM | 8.578681 | 3.217215 | 22.87499 | 1.75E-05 |
|  | BIRC5 | 5.292526 | 2.223923 | 12.59523 | 0.000165 |
|  | CCNB2 | 7.550117 | 2.845017 | 20.03652 | 4.91E-05 |
|  | CDK1 | 11.83276 | 4.057608 | 34.50656 | 6.04E-06 |
|  | DLGAP5 | 5.680177 | 2.291896 | 14.07761 | 0.000176 |
|  | FOXM1 | 4.836378 | 2.041453 | 11.4578 | 0.000341 |
|  | RACGAP1 | 7.884279 | 2.970407 | 20.92705 | 3.39E-05 |
|  | TOP2A | 6.584669 | 2.640377 | 16.42109 | 5.29E-05 |
|  | TPX2 | 7.293869 | 2.896368 | 18.36801 | 2.48E-05 |

Table S6: Statistical significance of tumor stage plots of hub genes measured by one-way ANOVA.

| **Genes** | **GSE10927** | | **GSE19750** | | **GSE75415** | | **GSE76019** | | **GSE76021** | | **TCGA-ACC data** | |
| --- | --- | --- | --- | --- | --- | --- | --- | --- | --- | --- | --- | --- |
|  | **F** | ***P*-value** | **F** | ***P*-value** | **F** | ***P*-value** | **F** | ***P*-value** | **F** | ***P*-value** | **F** | ***P*-value** |
| **ASPM** | 4.254 | 0.030 | 1.433 | 0.308 | 3.551 | 0.072 | 1.013 | 0.415 | 3.014 | 0.102 | 6.939 | 0.001 |
| **BIRC5** | 0.069 | 0.974 | 0.682 | 0.618 | 1.224 | 0.364 | 2.353 | 0.113 | 2.621 | 0.182 | 3.368 | 0.034 |
| **CCNB2** | 3.430 | 0.054 | 0.472 | 0.713 | 0.932 | 0.471 | 0.916 | 0.456 | 3.788 | 0.043 | 4.844 | 0.009 |
| **CDK1** | 0.105 | 0.954 | 0.160 | 0.918 | 1.301 | 0.342 | 1.141 | 0.365 | 2.046 | 0.216 | 6.779 | 0.001 |
| **DLGAP5** | 0.723 | 0.580 | 3.283 | 0.092 | 4.750 | 0.057 | 1.549 | 0.243 | 2.269 | 0.221 | 4.170 | 0.014 |
| **FOXM1** | 0.203 | 0.891 | 0.677 | 0.606 | 1.623 | 0.270 | 3.209 | 0.056 | 3.783 | 0.098 | 7.569 | 0.001 |
| **RACGAP1** | 0.070 | 0.973 | 0.242 | 0.863 | 1.470 | 0.302 | 1.240 | 0.330 | 2.557 | 0.114 | 4.717 | 0.009 |
| **TOP2A** | 1.333 | 0.312 | 1.385 | 0.387 | 2.508 | 0.145 | 2.748 | 0.080 | 2.517 | 0.158 | 4.687 | 0.008 |
| **TPX2** | 0.021 | 0.996 | 0.597 | 0.640 | 2.254 | 0.163 | 1.220 | 0.337 | 3.193 | 0.098 | 5.232 | 0.005 |

Note: ANOVA: Analysis of Variance.

Table S7: Statistical significance of tumor grade plots of hub genes.

| **Genes** | **GSE10927** | | **GSE19750** | |
| --- | --- | --- | --- | --- |
|  | **Unpaired *t* test** | | **One-way ANOVA** | |
|  | ***t*** | ***P*-value** | **F** | ***P*-value** |
| **ASPM** | 4.089 | < 0.001 | 2.603 | 0.116 |
| **BIRC5** | 3.818 | 0.001 | 1.776 | 0.207 |
| **CCNB2** | 4.062 | 0.001 | 6.271 | 0.013 |
| **CDK1** | 4.653 | < 0.001 | 3.193 | 0.072 |
| **DLGAP5** | 5.420 | < 0.001 | 3.291 | 0.071 |
| **FOXM1** | 3.530 | 0.002 | 1.152 | 0.374 |
| **RACGAP1** | 4.627 | < 0.001 | 3.504 | 0.060 |
| **TOP2A** | 3.881 | 0.001 | 0.572 | 0.645 |
| **TPX2** | 3.468 | 0.002 | 2.426 | 0.128 |

Note: ANOVA: Analysis of Variance.

Table S8: Expression levels comparison between ACC, ACA, and normal tissue.

| **Genes** | **GSE10927** | | **GSE12368** | | **GSE19750** | | **GSE75415** | |
| --- | --- | --- | --- | --- | --- | --- | --- | --- |
|  | **One-way ANOVA** | | **One-way ANOVA** | | **Unpaired *t* test** | | **One-way ANOVA** | |
|  | **F** | ***P*-value** | **F** | ***P*-value** | ***t*** | ***P*-value** | **F** | ***P*-value** |
| **ASPM** | 63.173 | < 0.001 | 6.275 | 0.016 | 4.316 | < 0.001 | 9.059 | 0.007 |
| **BIRC5** | 67.198 | < 0.001 | 6.012 | 0.013 | 2.936 | 0.005 | 6.417 | 0.018 |
| **CCNB2** | 106.467 | < 0.001 | 6.903 | 0.009 | 5.525 | < 0.001 | 13.865 | 0.002 |
| **CDK1** | 64.692 | < 0.001 | 8.410 | 0.005 | 5.663 | < 0.001 | 9.804 | 0.005 |
| **DLGAP5** | 54.137 | < 0.001 | 4.709 | 0.022 | 4.219 | < 0.001 | 9.379 | 0.005 |
| **FOXM1** | 79.337 | < 0.001 | 6.523 | 0.011 | 4.466 | < 0.001 | 12.030 | 0.001 |
| **RACGAP1** | 125.528 | < 0.001 | 7.384 | 0.005 | 6.331 | < 0.001 | 15.819 | 0.001 |
| **TOP2A** | 62.208 | < 0.001 | 4.582 | 0.027 | 4.815 | < 0.001 | 9.656 | 0.005 |
| **TPX2** | 88.707 | < 0.001 | 11.613 | 0.001 | 5.922 | < 0.001 | 17.453 | 0.001 |

Note: ACC: adrenocortical carcinoma; ACA: adrenocortical adenomas; ANOVA: Analysis of Variance.
